# Genome Wide Analysis of the Apple MYB Transcription Factor Family Allows the Identification of *MdoMYB121* Gene Confering Abiotic Stress Tolerance in Plants

**DOI:** 10.1371/journal.pone.0069955

**Published:** 2013-07-26

**Authors:** Zhong-Hui Cao, Shi-Zhong Zhang, Rong-Kai Wang, Rui-Fen Zhang, Yu-Jin Hao

**Affiliations:** 1 State Key Laboratory of Crop Biology, Shandong Agricultural University, Tai-An, Shandong, China; 2 MOA Key Laboratory of Horticultural Crop Biology and Germplasm Innovation, Shandong Agricultural University, Tai-An, Shandong, China; 3 College of Horticulture Science and Engineering, Shandong Agricultural University, Tai-An, Shandong, China; RIKEN Plant Science Center, Japan

## Abstract

The MYB proteins comprise one of the largest families of transcription factors (TFs) in plants. Although several *MYB* genes have been characterized to play roles in secondary metabolism, the MYB family has not yet been identified in apple. In this study, 229 apple *MYB* genes were identified through a genome-wide analysis and divided into 45 subgroups. A computational analysis was conducted using the apple genomic database to yield a complete overview of the MYB family, including the intron-exon organizations, the sequence features of the MYB DNA-binding domains, the carboxy-terminal motifs, and the chromosomal locations. Subsequently, the expression of 18 *MYB* genes, including 12 were chosen from stress-related subgroups, while another 6 ones from other subgroups, in response to various abiotic stresses was examined. It was found that several of these *MYB* genes, particularly *MdoMYB121*, were induced by multiple stresses. The *MdoMYB121* was then further functionally characterized. Its predicted protein was found to be localized in the nucleus. A transgenic analysis indicated that the overexpression of the *MdoMYB121* gene remarkably enhanced the tolerance to high salinity, drought, and cold stresses in transgenic tomato and apple plants. Our results indicate that the *MYB* genes are highly conserved in plant species and that *MdoMYB121* can be used as a target gene in genetic engineering approaches to improve the tolerance of plants to multiple abiotic stresses.

## Introduction

MYB TFs are widely distributed in all eukaryotic organisms, and these proteins comprise a large family of plant TFs, the members of which perform a variety of functions in plant biological processes [1], [2]. The MYB TFs are classified into three subgroups according to their MYB domain arrangement: R1R2R3, R2R3, and MYB-related (which contain a single MYB-like domain to recognize the major groove of DNA) [3], [4]. The major MYB TFs are the R2R3 MYB types, which have a modular structure with an N-terminal DNA-binding domain (the MYB domain) and an activation or repression domain that is usually located at the C terminus [3].

An increasing number of plant MYB TF members have been identified and characterized in numerous plant species based on the highly conserved DNA-binding domains in *Arabidopsis* (126 R2R3 MYB, five 3RMYB, and one 4R-like members), *Populus trichocarpa* (192 R2R3 MYB and five 3RMYB members), *Cucumis sativus* (55 R2R3 MYB members), and soybean (244 R2R3 MYB, six R1R2R3 MYB, and two 4R-like MYB members) [1], [5]–[7]. The 126 members of the R2R3 MYB family in *Arabidopsis thaliana* have been divided into subgroups [3]. New R2R3 MYB subgroups were identified according to comparative phylogenetic studies in other plant species such as in poplar and grapevine for which there are no representatives in *Arabidopsis thaliana* [3]. The expansion of the *R2R3 MYB* gene family in plants fits well with the observation that many R2R3 MYB TFs play important roles in plant-specific processes [3], [8].

In the past decade, the *MYB* genes, especially *R2R3 MYBs*, have been reported to be involved in diverse plant processes, including hormone signaling, cell cycle control, secondary metabolism, cellular morphogenesis, and meristem formation [8], [9]. For example, the overexpression of *AtMYB75/PAP1* and *AtMYB90/PAP2* results in the accumulation of anthocyanins in *Arabidopsis* [10], [11]. The R2R3 MYB proteins of subgroup 12 regulate glucosinolate biosynthesis, whereas *AtMYB28*, *AtMYB29*, and *AtMYB76* regulate the biosynthesis of aliphatic glucosinolates in aerial issues [12], [13]. *PhMYB1*, *AmMYBML2*, and *AtMYB16* are able to induce changes in the epidermal cell shape [14].

In addition, some R2R3 MYB members have also been shown to regulate plant responses to biotic and abiotic stress conditions [3]. For example, *AtMYB96* acts through the ABA signaling pathway to induce pathogen resistance by promoting salicylic acid biosynthesis and thus regulating stomatal movement, drought tolerance, and disease resistance in *Arabidopsis* [15], [16]. An R2R3-type MYB TF that is encoded by *Hos10* is required for cold acclimation in *Arabidopsis* plants [17]. The overexpression of the rice *OsMYB4* gene increases the chilling and freezing tolerance in transgenic *Arabidopsis thaliana* plants [18]. The ectopic expression of the apple *MdMYB10* gene in *Arabidopsis* enhances tolerance to osmotic stress [19]. Transgenic *Arabidopsis* plants that contain *FLP* and *AtMYB88* elevate their tolerance to abiotic stress by restricting the divisions that occur late in the stomatal cell lineage [20].

Large numbers of MYB proteins have been characterized through genetic approaches. There has been much effort devoted to the identification of R2R3 MYB in response to abiotic stresses in the model plants *Arabidopsis*, rice, and other species. Although several MYB genes with roles in secondary metabolism have been characterized in apple and other fruit trees [21], no previous study of this family has been conducted in apple. The draft genome sequence of the apple has been released (http://genomics.research.iasma.it/) [22], [23]. In this study, a genome-wide analysis was conducted based on the conserved DNA-binding domain to identify the *MdoMYB* gene models, the phylogenetic relationship of the MdoMYB proteins with other MYBs from different plant species, the genomic structure, the chromosome localization and other structural features. The expression patterns of twenty members in response to abiotic stresses were analyzed through real-time quantitative RT-PCR. Subsequently, we isolated an *R2R3 MYB* gene *MdoMYB121* due to its stress-induced expression and high similarity to other stress-related R2R3 MYBs from other species. Furthermore, its function was characterized in transgenic tomato and apple plants. Finally, a further exploration of the value of the *R2R3 MYB* function and the potential uses of the *MdoMYB121* gene in the improvement of the resistance of transgenic plants to abiotic stresses are discussed.

## Results

### Identification of Apple *MdoMYB* Genes

To identify the MYB-encoding genes in the apple genome, all known *Arabidopsis MYB* gene sequences were used as queries in multiple database searches against the proteome and genome files that were downloaded from the Apple GFDB database (Apple Gene Function and Gene Family Database: http://www.applegene.org/) and GDR database (Genome Database for Rosaceae: http://www.rosaceae.org/). Approximately 300 sequences that contain MYB or MYB-like repeat genes were identified in the apple genome. To confirm the putative *MYB* gene models that were identified, all of the genes derived from the selected *MdoMYB* candidate genes were examined using the domain analysis programs Pfam and SMART with the default cutoff parameters. In addition, we analyzed the domains of all of the apple peptide sequences through an HMM (Hidden Markov Model) analysis using Pfam. As a result, 222 typical R2R3 MYB proteins, five R1R2R3 MYB proteins, and two 4R-like MYB proteins were confirmed from the original data. This number is 1.7-fold higher than that found in *Arabidopsis thaliana*. To distinguish the remaining *MYBs*, we provisionally named them *MdoMYB1* through *MdoMYB229* based on their location on the chromosome, which was identified from the apple genome browser (Table S1). Among these MdoMYBs, the predicted proteins MdoMYB39, MdoMYB43, MdoMYB92, MdoMYB138 and MdoMYB190 belong to the R1R2R3 MYB family. In addition, MdoMYB175 and MdoMYB176 belong to the 4R-like MYB protein family. Additionally, we include the gene identifier, the genomic position, the pI (isoelectric point), the ORF length, the amino acid length, and the synonym in Table S1. As shown in this table, all of the identified *MYB* gene models encode proteins ranging from 126 (MdoMYB17) to 1710 (MdoMYB112) amino acids, and have a protein pI ranging from 4.61 (MdoMYB34) to 10.29 (MdoMYB181).

### Phylogenetic and Intron-Exon Structure Analysis of the Apple *MYB* Gene Family

To evaluate the evolutionary relationships within the *MYB* gene family, we performed a combined phylogenetic analysis of the *Arabidopsis* (132 members) and apple (229 members) MYB proteins using the NJ (neighbor-joining) method of the MEGA5 program (Figure S1). Furthermore, the peach and pear MYB families, which have 121 and 167 members, respectively, were also combined into the phylogenetic tree. The result showed high similarity among the Rosaceae MYBs (Figure S2). Based on sequence similarity and topology, we subdivided the apple MYB protein family into 45 subgroups, which were designated S1 through S25, H1 through H15, and performed a bootstrap analysis with 1,000 replicates for support (Figure S1A). As shown in Figure S1, all R2R3 MYB subgroups clustered separately from R1R2R3 and 4R MYB members. In our subgroup classification of the MYB proteins, we also took into account the results of Kranz et al. [4], Yanhui et al. [5], and Dubos et al. [3]. In addition, the functions of known *AtMYBs* are annotated (Figure S1B and references are shown in Text S2). Subgroups such as S1, S4, S11, S14, S20, S18, S21, S22, and H17 contained *Arabidopsis* MYB TFs which are known to be involved in the responses to abiotic stresses (Figure S1B).

We found that it was common to find that two or more MdoMYBs appeared to be putative orthologs of a single protein in *Arabidopsis*; for example, the phylogeny of subgroup H7 included only one AtMYB and five MdoMYBs. In contrast, six and three AtMYBs were included in subgroup S12 and S19, respectively. In addition, the phylogeny of subgroup S14 has the largest numbers of MYBs, including 14 MdoMYBs and 6 AtMYBs; subgroup S10, S19, H2, H8, H13, H14, H15, and H18 included the least number of MYBs.

In addition, the results of the intron-exon structure identification of the *MdoMYB* gene models are shown in Figure S3. To determine the numbers and positions of the exons and introns within each apple *MYB* gene model, we compared the full-length cDNA sequences with the corresponding genomic DNA sequences of the *MYB* distributed by introns. Only 7% of the *MYBs* had no introns in the coding region, whereas the remaining genes had up to 20 introns (*MdoMYB190*) based on their relative positions and phases.

### Sequence Features, Chromosomal Distribution and Duplication Events of the Apple *MYB* Gene Models

**Figure 1:**
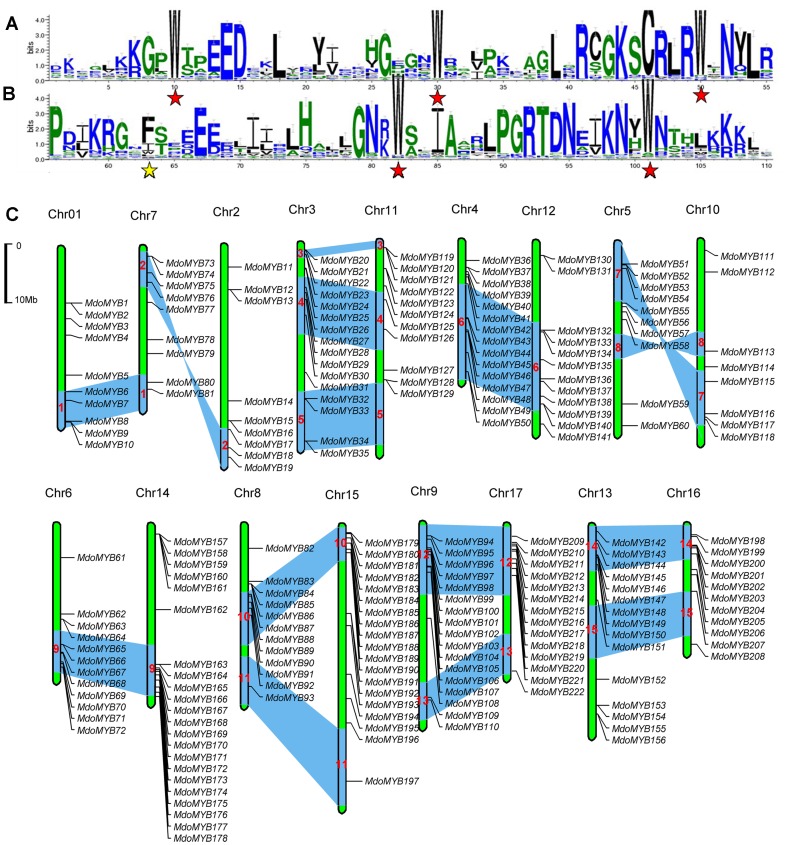
The DNA-binding domains of MdR2R3 MYB proteins and chromosomal locations for *MdoMYB* genes. The sequence logs of the R2 (A) and R3 (B) MYB repeats are generated by submitting the multiple alignments of all MdR2R3 MYB domains with ClustalX to the website of WebLogo software, which is used to analysis conserved residues (http://weblogo.berkeley.edu/logo.cgi). The overall height of each stack indicates the conservation of the sequence at that position and the bit score indicates the relative frequency of the corresponding acid. The conserved tryptophan residues (Trp) in the MYB domain are marked with red asterisks. The replaced residues in the R3 repeat are shown by yellow asterisks. (C) The chromosomal position of each *MdoMYB* gene was mapped according to the apple genome. The chromosome number is indicated at the top of each chromosome. The scale is in megabases (Mb). Blue bars on the chromosomes and red numbers inside the blue bars indicate the predicted 15 duplication regions.

To investigate the DNA-binding domains of the apple MYB proteins, logos were generated using WebLogo, which is designed to show the conservation at a particular positions within a multiple sequence alignment [24]. The results showed that the R2 and R3 repeats of MdoMYBs contain conserved amino acids, especially characteristic Trp residues (Figure 1A–B). Among these, the R2 repeat contains three conserved Trp residues. The R3 repeat also exhibited a high conservation of the second and third Trp residues, whereas the first Trp was generally replaced by Phe or Ile. Subsequently, MEME software was used to identify the structural similarities of C-terminal motif. It was found that, among 45 subgroups analyzed, 27 ones possessed one to four identical C-terminal motives in each subgroup, while the other 18 did not at all (Figure S1C).

The information concerning the expansion events of the *MYB* gene family in apple remain unclear so far. Therefore, the chromosomal locations of the *MdoMYBs* were retrieved from the apple genome data that were downloaded from the GDR database. The analyses of the genome chromosomal locations revealed that the apple *MYB* genes are distributed on all chromosomes. Although each of the 17 apple chromosomes contained *MYBs*, the distribution appeared to be uneven (Figure 1C). Seven gene models (*MdoMYB223*, *224*, *225*, *226*, *227*, *228*, and *229*) could not be conclusively mapped to any chromosome. The gene density per chromosome ranged from 4.1% to 9.9%; the highest density was observed on chromosome 14 (22 members), and the lowest density was found on chromosome 2 (nine members). In general, the middle part of the chromosomes exhibited a low density of *MYB* genes, whereas the ends exhibited a relatively high density. Also, large-scale segmental duplication events were investigated. It was found multiple pairs linked each of at least 15 potential chromosomal/segmental duplications (Figure 1C, pairs of bars with numbers 1 through 15 in the blue areas), such as the large sections on chromosomes 3 and 11.

### Expression Profiles for 20 *MdR2R3 MYB* Genes in Response to Abiotic Stresses

**Figure 2:**
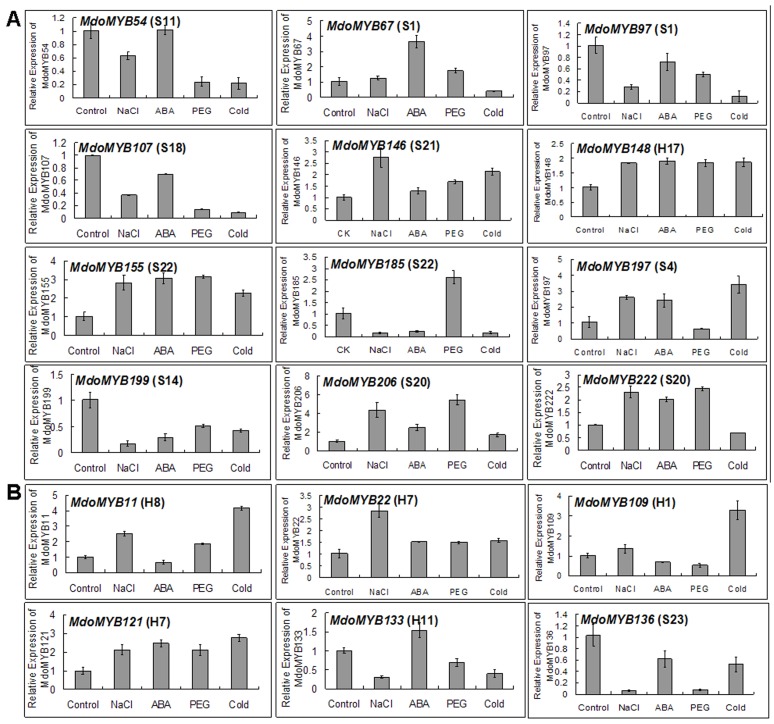
Expression analysis of 18 *MdR2R3 MYB* genes under abiotic stress treatments. The expression pattern of 18 *MdR2R3 MYB* genes in response to NaCl, ABA, PEG, and cold treatments at 2 h, 12 were chosen from stress-related subgroups (A) and 6 ones from other subgroups (B). *MdACTIN* was used as an internal standard.

Mounting evidence suggests that the *R2R3 MYB*s play important roles in abiotic stress tolerance in various plant species. To examine if the expression of apple *R2R3 MYB* genes is induced by abiotic stresses including NaCl, ABA, PEG, and cold treatments, 12 apple *R2R3 MYB* gene models (*MdoMYB54*, *67*, *97*, *107*, *146*, *148*, *155*, *185*, *197*, *199*, *206*, and *222,* Figure 2A) were chosen from stress-related subgroups, while another 6 ones (*MdoMYB11*, *22*, *109*, *121*, *133*, 136, Figure 2B) from other subgroups. These gene models represented really genes as demonstrated by EST blast through the GenBank database and sequencing of the corresponding RT-PCR production (Text S1).

Subsequently, real-time RT-PCRs were performed to analyze their expressions in response to four treatments, i.e. NaCl, ABA, PEG, and cold (Figure 2, and primers are listed in Table S2). The results showed that the transcript levels of *MdoMYB22*, *121*, *146*, *148*, *155*, and *206* increased in response to the four treatments. The levels of *MdoMYB11*, *67*, *109*, *197*, and *222* were enhanced in response to only two or three of the treatments. The expressions of *MdoMYB133* and *MdoMYB185* were induced by ABA, and PEG, respectively, but inhibited by the other treatments. In contrast, the transcription levels of *MdoMYB54, 97*, *107*, *136*, and *199* were downregulated by these treatments.

### Seed Germination and Seedling Growth are Insensitive to High Salinity in *MdoMYB121* Transgenic Tomato

**Figure 3:**
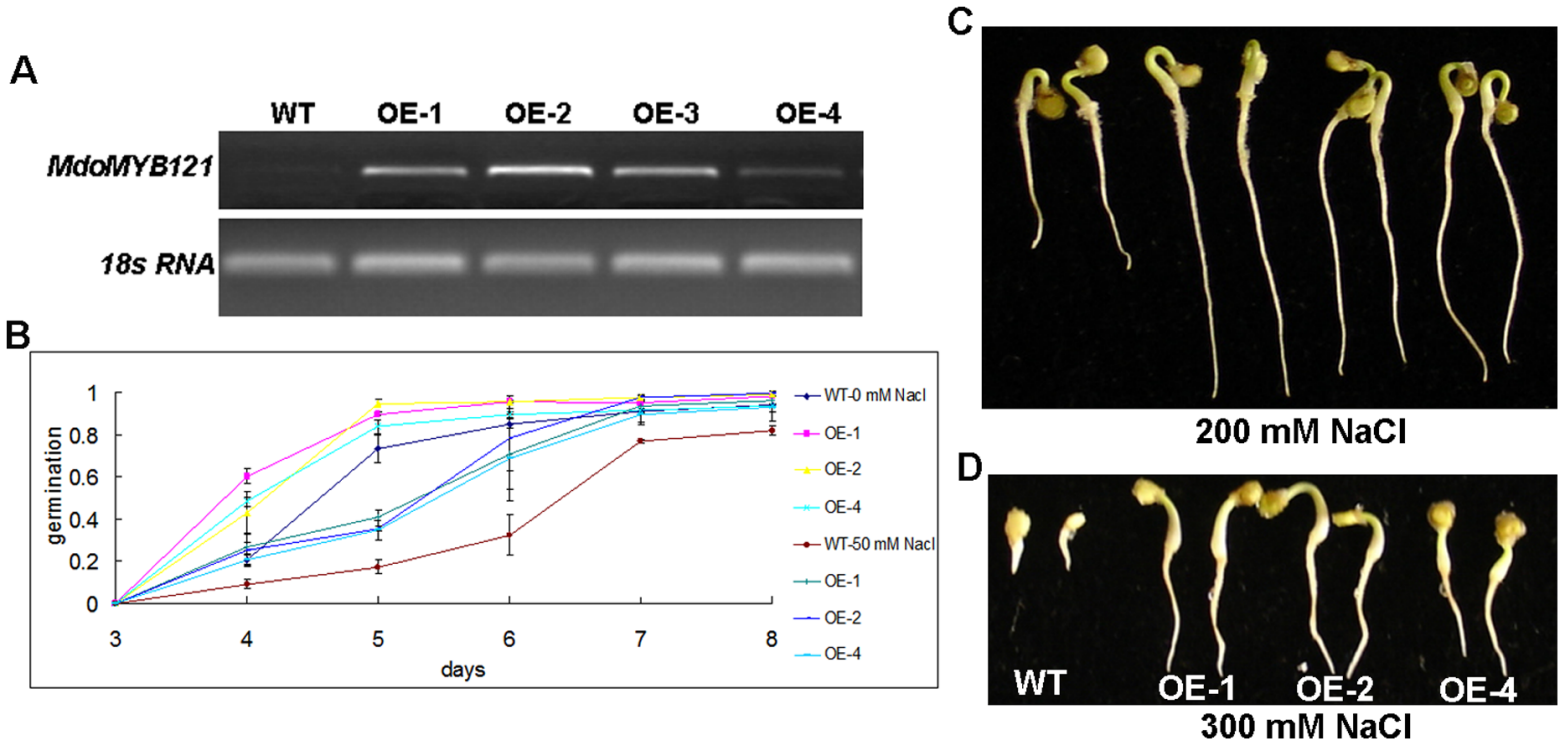
*MdoMYB121* transgenic tomato seed germination and seedling development is more tolerant to NaCl. (A) Expression levels of *MdoMYB121* in independent transgenic tomato. *MdACTIN* was used as an internal standard. (B) Seed germination on MS or MS supplemented with 50 mM NaCl of transgenic *MdoMYB121* and the WT. All tests were repeated at least three times, and approximately 50 seeds were counted for each experiment. Data are expressed as the means ± SE. (C-D) WT and transgenic *MdoMYB121* plants were germinated on MS medium and then transferred to a new MS medium supplemented with different concentrations of NaCl for 15 days.

MdoMYB121 (Genebank accession number KC834015) was subgrouped into H7 R2R3 MYB subgroup which does not contain known stress-related *MYB* gene (Figure S1). However, its expression was remarkably induced by the four abiotic stress treatments imposed (Figure S4). The phylogenetic tree further demonstrated that MdoMYB121 exhibited similarity to different extents to abiotic stress-related MYBs from other species (Figure S5). Also, MdoMYB121-GFP fusion protein is subcellularly localized in nucleus, just like other transcription factors (Figure S6). Therefore, MdoMYB121 was chosen for functional characterization. *MdoMYB121* gene was isolated from apple cDNA template and then genetically transformed into tomato. Three lines, named OE-1, OE-2, and OE-4, were used for further investigation (Figure 3A). The *MdoMYB121* transcripts showed high levels in the functional leaves of the three selected transgenic lines, but no transcripts were detected in the wild-type tomato using primers specific for *MdoMYB121*. Then, the seed germination and young seedling growth were examined to determine their response to high salinity. There was no apparent difference in the seed germination between the WT and the transgenic plants under normal growth conditions. Compared with the WT, the *MdoMYB121* plants exhibited significantly higher seed germination in response to 50 mM NaCl (Figure 3B). For example, approximately 70% of the *MdoMYB121*-overexpressing seeds germinated at day 6 compared with 30% of the WT seeds.

Subsequently, the effects of the ectopic expression of *MdoMYB121* on tomato seedlings under salt stress were determined. The transgenic and WT seedlings pre-germinated on MS medium and then transferred to media containing 200 mM and 300 mM NaCl. Under 200 mM NaCl, the transgenic seedlings formed longer roots and exhibited faster development of green cotyledons than the WT, although the growth of both the WT and the transgenic seedlings was significantly inhibited (Figure 3C). When the concentration of the NaCl was increased to 300 mM, the growth of the WT was completely inhibited, and the cotyledons could not form normally. However, the transgenic seedlings formed longer roots, and some of them developed green cotyledons and continued to grow (Figure 3D).

These results suggest that the overexpression of *MdoMYB121* confers an increased tolerance to salt stress during seed germination and early seedling development.

### Ectopic Expression of *MdoMYB121* Confers Enhanced Tolerance to Abiotic Stresses in Tomato

**Figure 4:**
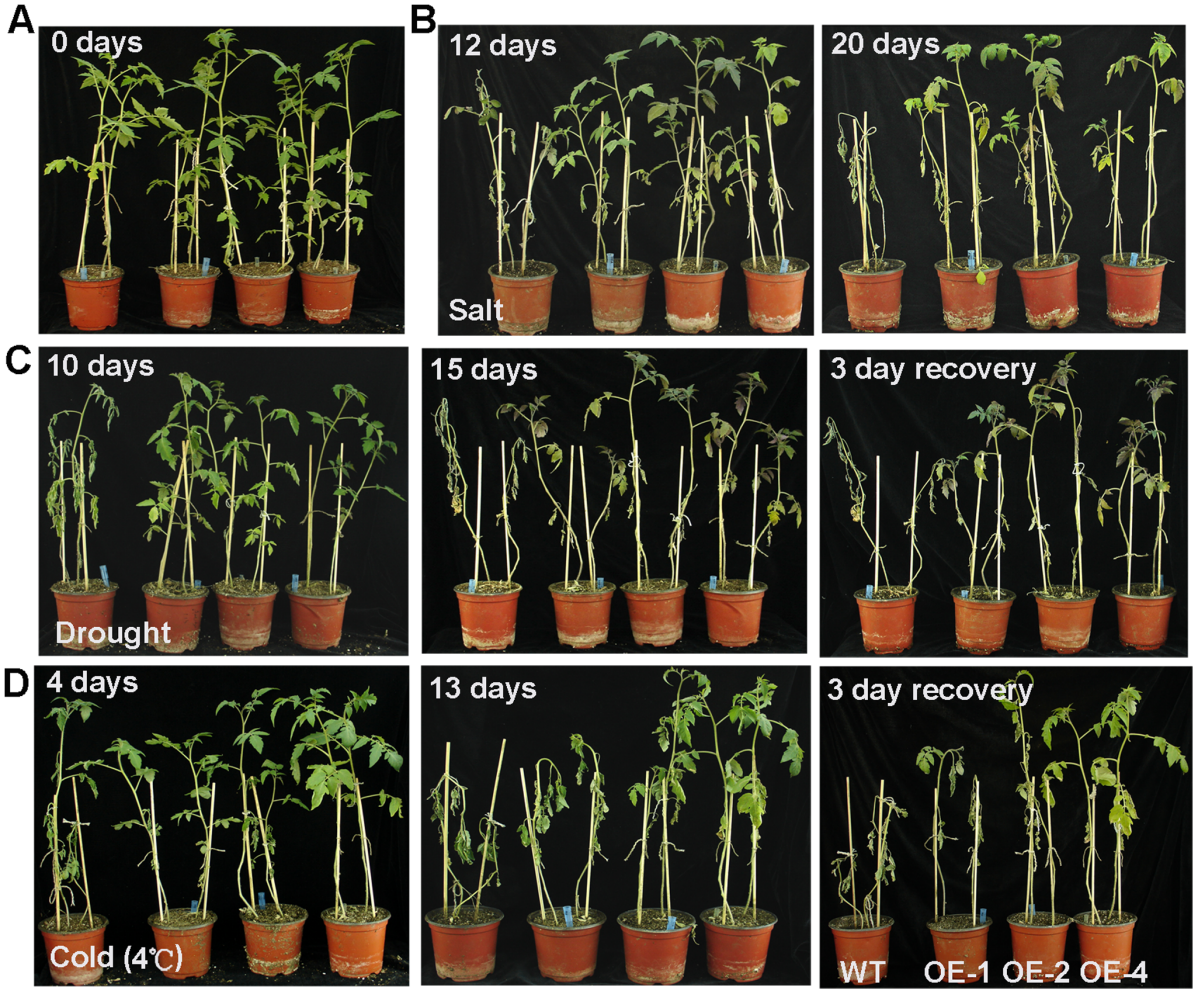
Abiotic stress tolerance of *MdoMYB121* transgenic tomato plants. (A–D) Tolerance of transgenic tomato plants under normal conditions, 300 mM NaCl for 20 d, dehydration for 15 d with a 3 d recovery, 4°C for 13 d with a 3 d recovery.

We then tested the influence of the ectopic expression of *MdoMYB121* on adult tomatoes exposed to salt, drought, and cold. All of the adult plants perform well, and there were no significance differences between the WT and the transgenic tomato plants under normal growth conditions (Figure 4A). The transgenic tomato plants grew well when irrigated with 300 mM NaCl solution for 12 days, whereas the WT plants grew poorly and exhibited chlorosis. After twenty days, the WT plants died. In contrast, the leaves of the transgenic plants turned yellow and wilted significantly, but continued to grow (Figure 4B).

**Figure 5:**
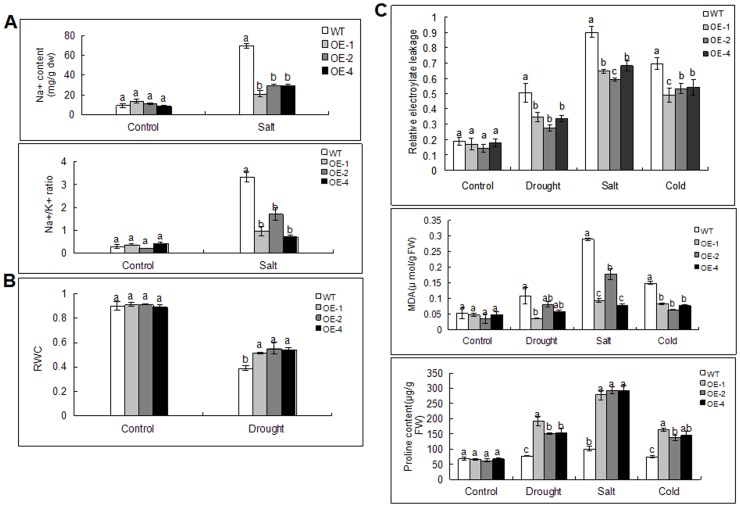
Physiological index of tomato plants under abiotic stresses. (A–B) Na^+^ content and Na^+^/K^+^ ratio under 300 mM salt stress for 20 d and relative water content of leaves under dehydration for 15 d of WT and *MdoMYB121* transgenic tomato plants. (C) Relative electrolyte leakage, MDA and proline content under abiotic stresses of WT and *MdoMYB121* transgenic tomato plants. Data are expressed as the means ± SE.

To elucidate why transgenic plants are more tolerant to high salinity than the WT plants, the Na^+^ content and the Na^+^/K^+^ ratio in the functional leaves were measured under salt stress. The results show that the transgenic lines accumulated a lower Na^+^ content and a lower Na^+^/K^+^ ratio than the WT plants, which indicates that *MdoMYB121* is involved in the regulation of the physiological balance between Na^+^ and K^+^ in response to salt stress (Figure 5A).

For the dehydration tolerance assay, transgenic *MdoMYB121* and WT tomato plants were withheld from water for 15 days and then re-watered for 3 days (Figure 4C). After 10 days, the transgenic plants grew well under normal growth, whereas the WT plants wilted. After 15 days, almost all of the WT had died, and all transgenic plants were adversely wilted. However, the growth of the transgenic plants recovered after re-watering, whereas the WT plants did not.

To estimate the water loss under drought conditions, the relative water content (RWC) in WT and transgenic *MdoMYB121* plants were measured. The results indicate that the transgenic lines exhibit a greater potential to maintain water than the WT plants (Figure 5B), which indicates that the overexpression of *MdoMYB121* enhances drought tolerance at least partially by reducing the water loss.

Tomato plants were exposed to 4°C to test whether the *MdoMYB121* ectopic expression enhances cold tolerance. After 4 days of the cold treatment, the WT plants were adversely affected and wilted, but the transgenic plants appeared normal in morphology (Figure 4D). Most of the WT plants died after 13 days, whereas the transgenic lines were significantly wilted but performed better. Subsequently, the transgenic plants and WT were transferred to normal conditions for a 3-day recovery period. The results show that the growth of the transgenic plants completely recovered, although the leaves were shrivelled and yellow, but the WT plants died.

To investigate the physiological mechanisms underlying the function of *MdoMYB121* in response to abiotic stresses, the contents of the membrane damage and the intracellular oxidative stress responsive index, such as the relative electrolyte leakage and the MDA and proline levels, were measured [25]–[27]. The results show that the transgenic *MdoMYB121* tomato plants exhibit lower levels of electrolyte leakage and MDA and an elevation in the content of the osmoprotectant proline in response to salt, drought, and cold stresses (Figure 5C). We hypothesize that *MdoMYB121* reduces the membrane damage and promotes the accumulation of protective compounds against environmental stress.

### *MdoMYB121* Overexpression Enhances Tolerance to Abiotic Stresses in Transgenic Apple Rooted Plantlets

**Figure 6:**
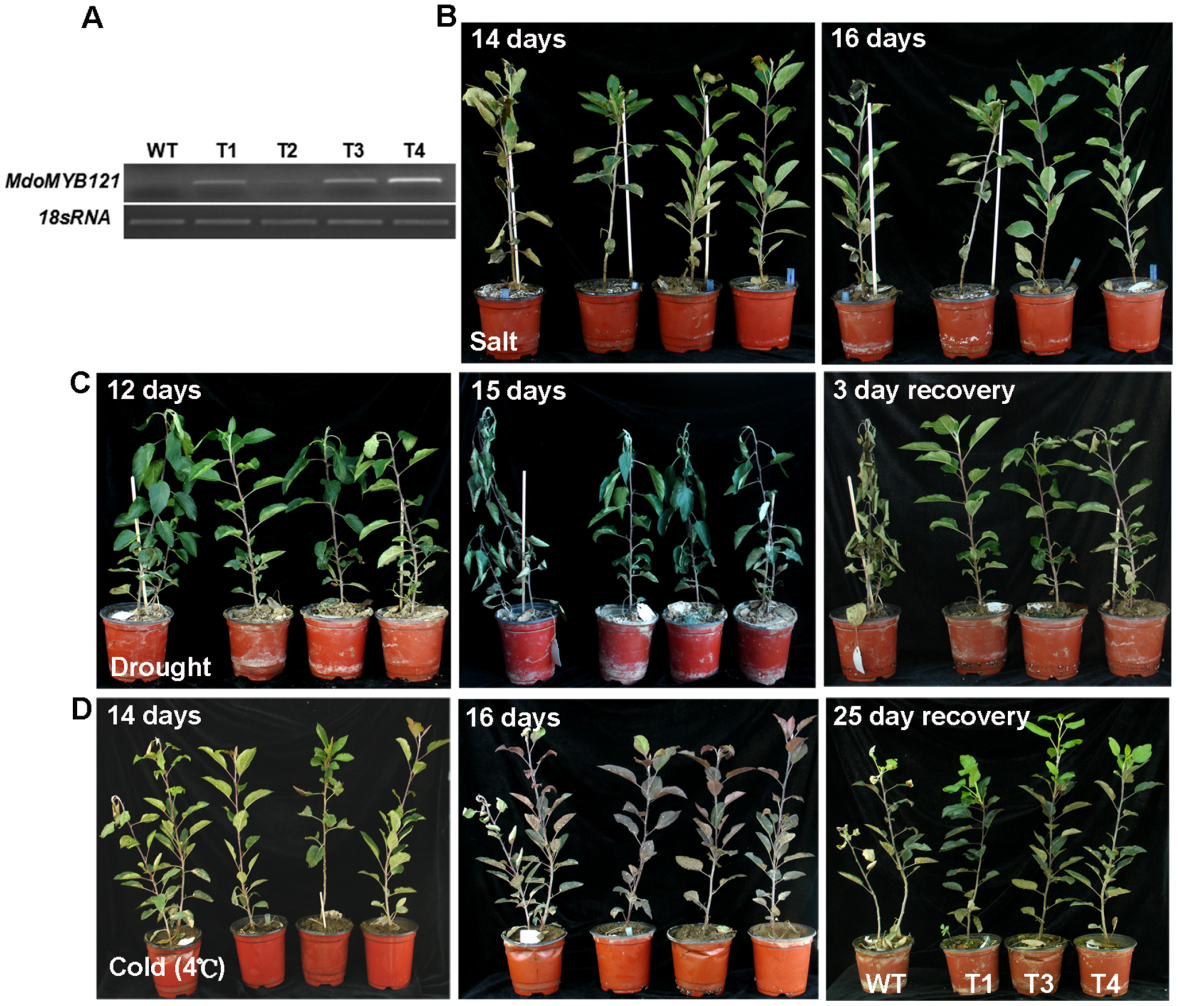
*MdoMYB121* overexpression enhances tolerance to abiotic stresses in apple plantlets. (A) Expression levels of *MdoMYB121* in independent transgenic apple lines. (B–D) Tolerance of transgenic rooting apple plantlets for 300 mM NaCl for 16 d, dehydration for 15 d with a 3 d recovery and 4°C for 16 d with a 25 d recovery.

The performance of the *MdoMYB121* transgenic tomato plants under abiotic stresses indicate that this gene might also operate in apple. To confirm that *MdoMYB121* really confers tolerance to salt, drought, and cold stress in apple, transgenic apple plants carrying *MdoMYB121* were produced. Three transgenic lines of each *MdoMYB121* (T1, T2, and T4) were chosen for further investigation (Figure 6A). In the tolerance assays, six-month-old transgenic self-rooted plantlets were used.

For the analysis of salt tolerance, the transgenic and WT plantlets were irrigated with 300 mM NaCl solution for 16 days. After 14 days, the leaf color of the *MdoMYB121* lines were normal, whereas the WT controls were discolored, which indicates that the leaf necrotic damage of the WT control was more serious than that found in the transgenic lines (Figure 6B). After 16 days, almost all of the WT plants died, whereas the transgenic plants performed better, although some leaves, especially the young leaves, were discolored. These results suggest that the *MdoMYB121* overexpression confers enhanced salt tolerance in the transgenic apple plants.

For the drought tolerance assay, the WT and transgenic rooted plantlets were deprived of water for 15 days and then re-watered for 3 days. The results show that the WT plants wilted after being deprived of water for 12 days and that the transgenic lines performed well (Figure 6C). After 15 days, the WT plants exhibited a noticeable loss of color, brittle phenotypes, and even death, whereas the transgenic apple plants were inhibited to a lesser extent. After relief of the water deficit stress for 3 days, the transgenic lines demonstrated a recovery phenotype, but the WT plants died. These results demonstrate that the *MdoMYB121* transgenic apple plants exhibit noticeably enhanced tolerance to drought stress treatment.

The cold stress tolerance of the *MdoMYB121* apple plants was then tested. Compared with the *MdoMYB121* plants, the WT plants had a significantly wilted phenotype in the leaves after exposure to 4°C for 14 days (Figure 6D). After 16 days, the leaves of all of the WT and transgenic apple plants turned red, but the WT plants exhibited more serious necrotic damage compared with the transgenic lines. Finally, the WT and transgenic apples were returned to normal conditions at 22°C for an additional 25 days. We observed that the growth of all of the plants recovered, but the transgenic plants grew better compared with the WT. Therefore, *MdoMYB121* overexpression enhances cold tolerance in transgenic apple plants.

## Discussion

The MYB transcription family is large, functionally diverse, and represented in all eukaryotes. It is one of the most abundant transcription factor families in plants and has been identified in *Arabidopsis*, rice, maize, soybean, and grape [1], [7], [28], [29]. In this study, we identified 229 apple *MYB* gene models from the apple genome sequence, which contribute to a large plant MYB TF family. Furthermore, the overexpression of an *R2R3 MYB* gene *MdoMYB121* enhances tolerance to abiotic stresses in both tomato and apple plants.

Compared with MYB proteins in various plant species, the MYBs that tended to cluster together were usually from the same lineage, which indicates recent common evolutionary origins. In this study, putative orthologs of a single AtMYB protein with two or more apple MYBs were found, which indicates that the apple MYBs experienced duplications after their divergence from *Arabidopsis*. The occurrence of gene duplication throughout plant evolution has long been recognized and contributes to the expansion of the apple *MYB* genes and the establishment of the new gene functions. In this study, *MdoMYB121* belongs to the subgroup H7 and exhibits similarity to *AtMYB5*, which has been characterized to have functions in seed coat differentiation and is partially redundant with *AtMYB123* in the regulation of tannin [3]. Therefore, the response to abiotic stresses of *MdoMYB121* expands the function of this subgroup, and *AtMYB5* may be related to stress induced responses.

In general, the gene functions of a subgroup appear to be similar. For example, the *MYBs* in subgroups S6 and S15 are needed for anthocyanin biosynthesis and the regulation of trichome development in *Arabidopsis*, respectively. Therefore, not all members of the same subgroup perform an absolutely conserved function. In subgroup S4, *AtMYB32* is involved in the normal pollen development in *Arabidopsis thaliana* [3], whereas *AtMYB8* is required for freezing tolerance and its loss of function results in hypersensitivity to NaCl and increases the transcripts of stress-related genes [30].

Remarkably, the genes in the same group generally exhibit the same intron pattern, and the position of the intron is almost completely conserved within most subgroups [1]. Additionally, the first two exons of the modal lengths are very similar and highly conserved [31]. This finding tests the reliability of our phylogenetic analysis and constitutes an independent criterion. However, in our study, the members of *MdoMYBs* within the same subgroup did not always show similar intron-exon structures and formed a complex exon/intron organization in the entire ORF.

In general, MYB proteins are characterized by a highly conserved DNA-binding domain in the N-terminus [1], [3], [4], [32]. This domain mainly consists of up to four imperfect repeats (R) and contains three helices of each repeat, the second and third of which form a helix-turn-helix structure when bound to DNA [3], [4], [7]. There are R2 and R3 repeats among the 222 MdR2R3 MYB proteins. Three highly conserved Trp residues were generally contained in the R2 repeat, whereas the first Trp residue in the R3 repeat exhibits variability. The substitution at this first Trp residue may be responsible for the recognition of novel target genes and/or may significantly impair the DNA-binding activity [33]. In contrast, the other regions, especially the C-terminus, of the R2R3 MYB proteins are highly variable [3], [7]. However, the conserved C-terminal motifs of MYB proteins were identified in soybean, *Arabidopsis*, and rice [1], [4], [34]. In this study, it was found that some apple MYB proteins have conserved C-terminal motifs, suggesting that they maybe have similar functions. In addition, some MYB proteins have been identified from different species. They share an identical C-terminal W/Y-MDDIW motif which is important for their trans-activation activity [35]. Based on the conserved N-terminus and the transcriptional activation or repression in the C-terminal domain of the MYB TFs, these proteins play important roles in multiple plant physiological processes, such as tolerance to abiotic stresses.

During the evolution of plants, gene duplication events have long occurred. These contribute to the establishment of new gene functions and underline the origins of evolutionary novelty [36], [37]. Relatively recent genome-wide duplication (GWD) events and the expansion of gene families in the Pyreae resulted in the transition from nine ancestral chromosomes to 17 chromosomes [22]. In this study, it was found that 15 potential chromosomal/segmental duplications of multiple pairs are associated with genome-wide duplication in the apple genome, as has been reported by Velasco et al. [22], who supported the hypothesis that the large-scale expansion of the *R2R3 MYB* family in the apple genome evolved from putative duplication events. Furthermore, there exist several types of segment pairs during chromosomal duplication events. For example, they were classified into α, β, and γ type in *Arabidopsis* according to the relative time of duplication [38]. It has been previously reported that subgroup S12, which is responsible for regulate aliphatic or indolic glucosinolate biosynthesis in *Arabidopsis*, comes from a specific β-type duplication in the order Brassicales to adapt herbivory [5], [39], [40]. However, there is no apple, rice and grape MYB gene models grouped into S12, probably because β-type duplication does not occur in these species [5], [31]. In addition, many fragments have not yet been assigned to a particular chromosome on the draft genome sequences [22], and this may the reason why seven genes could not be conclusively mapped to any chromosome.

It has been well established that the MYB s play important roles in various life processes [2], [3], [15]. In fact, many *MYB* genes in a wide range of plant species, such as *Arabidopsis*, rice, and wheat, are involved in the response to abiotic stresses [3], [15], [41]. In *Arabidopsis*, the overexpression of *AtMYB44* and *OsMYB4* enhances tolerance to abiotic stresses [2], [18]. In wheat (*Triticum aestivum*), TaMYB33 exhibited high similarity with other stress-related MYBs. Its transcription is induced by abiotic stresses, and its overexpression increases the tolerance of the plant to abiotic stress [41]. In grape, VvMYB60 is identified to an ortholog of AtMYB60 according to a bioinformatic- genomic analysis, and is characterized to be involved in stomatal regulation and thus in abiotic stress responses [42]. However, little is known about the specific roles of *MYB* genes in apple in response to abiotic stresses; in fact, just a few of *MdMYB* genes is found to be involved in stress tolerance, such as *MdMYB10* [19]. Our genome-wide analysis and expression profiling of apple MYB TFs in response to multiple abiotic stresses provide a baseline for their further functional characterization with stress tolerance. Accordingly, *MdoMYB121* was chosen to further investigate whether it plays a role in the responses of plants to abiotic stresses, as many *R2R3 MYB* TFs do in other plant species [3]. It was found that *MdoMYB121* overexpression enhances tolerance to high salinity, drought, and cold in transgenic tomato and apple plants.

Plant salt tolerance is a complex trait. Multiple physiological and biochemical mechanisms are involved to maintain a high cytosolic K^+^ uptake, prevent excess Na^+^ accumulation in the plant symplast, and/or maintain desirable K^+^/Na^+^ ratios in the cytosol [43], [44]. *Thellungiella halophila* is a salt-tolerant relative of *Arabidopsis thaliana*. This plant exhibits extreme tolerance to high salinity through the support of K^+^/Na^+^ homeostasis in the root ion-channel [45]. A mutation in the *Salt Overly Sensitive 2* (*SOS2*) gene causes an Na^+^ and K^+^ imbalance that renders the mutant plants more sensitive to growth inhibition in high Na^+^ and low K^+^ environments [46]. Additionally, the transporter *AtHKT1* controls Na^+^ entry and high affinity K^+^ uptake in the leaves of *Arabidopsis* to determine its tolerance to salt stress [47]. In this study, *MdoMYB121* transgenic tomato plants accumulated less Na^+^ and maintained a lower Na^+^/K^+^ ratio under high salinity than the WT control plants, which shows that this gene enhances stress tolerance. The result suggests that *MdoMYB121* is involved directly or indirectly in the regulation of Na^+^ and K^+^ homeostasis in plant cells under salt stress.

During dehydration periods, plants accumulate the stress-response hormone abscisic acid (ABA), which induces the rapid closing of stomata to prevent water loss by transpiration [48]. The relative water content (RWC) indicates the water status of a plant at any given time because it closely reflects the balance between the water supply and the transpiration rate [48]. The R2R3 MYB proteins FOUR LIP (FLP) and MYB88 restrict divisions late in the stomatal cell lineage in *Arabidopsis* [49]. The *flp-1 myb88* double mutant is much more susceptible to drought and shows increased rates of water loss [20]. The overexpression of *AtMYB61* can minimize the water loss in response to drought stress and might be employed as a strategy for the growth of crop plants in arid regions [50]. Transgenic plants overexpressing *OsMYB3R-2* exhibit a slower water loss in detached rosette leaves compared with WT, which indicates that this gene enhances drought tolerance [51]. In this study, *MdoMYB121* transgenic lines maintained a higher leaf RWC and therefore exhibited a higher drought tolerance than WT controls in response to drought stress.

In addition, abiotic stresses result in membrane damage, as indicated by the MDA concentration and the electrolyte leakage. In parallel, proline is accumulated to protect the membrane integrity [27], [52]–[54]. In previous reports, an elevated tolerance to osmotic and freezing stresses is accompanies by a lower MDA content and a higher proline accumulation in *MdMYB10* and *OsMYB4* transgenic plants, respectively [18], [19]. In addition, the *AtMYB2* transgenic *Arabidopsis* exhibits a higher tolerance to osmotic stress, as measured by the electrolyte leakage from cells [55]. In this study, transgenic *MdoMYB121* plants produced more proline and less MDA and exhibited less electrolyte leakage than the control plants, which indicates that these plants exhibited enhanced tolerance to multiple stresses.

Taken together, our results indicate that the *MYB* genes are highly conserved among different plant species. The *in silico* analysis performed provides a valuable method for further exploration of the functions of the *R2R3 MYB* genes in apple. As a result, *MdoMYB121* was functionally characterized as a target gene for genetic engineering approaches to improve the tolerance of fruit trees and other crops to multiple abiotic stresses.

## Materials and Methods

### Ethics Statement

No specific permits were required for the described field studies. The location is not privately-owned or protected in any way, and the field studies did not involve endangered or protected species.

### The Identification of *MYB* Genes in Apple

Two approaches were used to identify the members of the *MdoMYB* gene family in apples. First, all known *Arabidopsis MYB* gene sequences were used as queries to perform multiple database searches against the proteome and genome files that were downloaded from the Apple GFDB database (Apple Gene Function and Gene Family Database: http://www.applegene.org/) as well as the GDR database (Genome Database for *Rosaceae*: http://www.rosaceae.org/). Stand-alone versions of BLAST (Basic Local Alignment Search Tool: http://blast.ncbi.nlm.nih.gov) which are available from the NCBI, were used with an e-value cutoff of 1e-003. All of the protein sequences deriving from the selected *MdoMYB* candidate genes were examined with the domain analysis programs Pfam (Protein family: http://pfam.sanger.ac.uk/) and SMART (Simple Modular Architecture Research Tool: http://smart.embl-heidelberg.de/) with the default cutoff parameters. Second, we analyzed the domains of all of the apple peptide sequences using an HMM (Hidden Markov Model) analysis while searching Pfam. Then, we obtained the sequences by using the PF00249 Pfam number, which contained a typical MYB DNA-binding domain, from the apple genome sequences by making use of a Perl-based script. Finally, all of the protein sequences were compared with known MdoMYB sequences by applying Clustal X (http://www.clustal.org/) to verify that the sequences were candidate MdoMYBs.

The isoelectric points and protein molecular weights were obtained with the help of the proteomics and sequence analysis tools on the ExPASy proteomics server (http://expasy.org/). The chromosomal locations were found in the GDR database by using a Perl-based program.

In the same way, we obtained 167 *Pyrus sorotina MYB* genes according to the *Pyrus* genome (http://peargenome.njau.edu.cn/). Additionally, 121 *Prunus persica MYB* genes were obtained from the Genome Database for Rosaceae (GDR).

### Sequence Alignment and Phylogenetic Analysis

The MYB sequences were aligned using the Clustal X program with BLOSUM 30 as the protein weight matrix. The MUSCLE program (version 3.52) was also applied to perform multiple sequence alignments to confirm the Clustal X result. The phylogenetic trees for the MdoMYB protein sequences were constructed using the NJ (neighbor-joining) method of the MEGA5 program (http://www.megasoftware.net/) and the p-distance for the complete deletion option parameters. The reliability of the trees was tested using a bootstrapping method with 1,000 replicates. The images of the phylogenetic trees were drawn in MEGA5.

### The Chromosomal Location and Structure of the *MdoMYB* Genes

The chromosomal locations and gene structures were retrieved from the apple genome data that were downloaded from the GDR database. The remaining genes were mapped to the chromosomes with MapDraw, and the gene structures of the *MdoMYB* genes were generated with the GSDS software (http://gsds.cbi.pku.edu.cn/) [56].

### Protein Motif Identification

Motifs were detected within each subgroup using MEME version 3.5.7 tool to identify conserved motifs shared among MYB proteins [57]. The following parameter settings were used: distributing of motifs, zero or one per sequence; maximum number of motifs to find, 50; minimum width of motif, six; maximum width of motif, 117 (to identify long R2R3 MYB domains); and motif must be present in all members within the same subfamily.

### Plant Materials and Treatments

*In vitro* shoot cultures of apple ‘Gala’ were maintained on MS medium containing 0.5 mg L^−1^ 6-BA, 0.2 mg L^−1^ IAA, and 0.1 mg L^−1^ GA under long-day conditions (16-h light/8-h dark cycle) and then subcultured with a 4-week interval. Four-week-old shoot cultures were transferred to a root-inducing medium, i.e., MS medium containing 0.1 mg L^−1 ^IAA to obtain self-rooted plantlets. The rooted plantlets were then transferred to pots of nursery soil for further investigation.

Approximately 4-week-old apple shoot cultures were transferred to MS medium containing 200 mM NaCl, 4°C low temperature, 10% PEG, or 100 µM ABA. These were sampled after 0, 2, 4, 6, 12, and 24 h for the analysis of the gene expression in response to the stress treatments. The 5′ UTR-specific primers for the real-time quantitative RT-PCR analysis are listed in Table S2.

### Construction of *MdoMYB121* Overexpression Vector

The total RNA was isolated from *in vitro* shoot cultures of ‘Gala’ using the RNA plant plus Reagent (Tiangen, Beijing, China). Two micrograms of the total RNA was used to synthesize the first-strand cDNA for *MdoMYB121* gene cloning (Takara, Dalian, China). To generate *MdoMYB121*-overexpressing plants, the full-length cDNA of *MdoMYB121* was digested with *Xba*I/*Sal*I and cloned into the pBI121 vector, in which it was placed under the control of the *CAMV 35S* promoter. Then, *Agrobacterium tumefaciens* strain LBA4404 was transformed with the construct. The PCR products used for the constructs were amplified from the *MdoMYB121* cDNA using the following primers: 5′-GCGTCGACATGAGGAACCCATCG-3′ (forward) and 5′-GCTCTAGACTACTCCGTTTGATCATTAA-3′ (reverse).

### Subcellular Localization of MdoMYB121 Protein

The subcellular localization of MdoMYB121 protein was determined as described by Li et al. [58]. The *MdoMYB121* ORF without a stop codon was obtained by RT-PCR using the following primers containing digestion sites (underlined): 5′- GCTCTAGAATGAGGAACCCATCGTCTT-3′ (forward) and 5′-GCGGTACCCTCCGTTTGATCATTAACG-3′ (reverse). The PCR products were digested with *XbaI*/*KpnI* and cloned into the pBI121-GFP vector. Then, the p35S:MdoMYB121-GFP and the control vector p35S:GFP were introduced into *Agrobacterium* strain LBA4404 and transformed into onion epidermal cells. These cells were pre-incubated on MS medium plates under light at 22°C for 24 h, and the subcellular fused protein was detected by confocal microscope (Zeiss LSM 510 META, Jena, Germany).

### Tomato Transformation and Stress Tolerance Assay in Transgenic Tomato

Tomato (*Solanum lycopersicum* L. cv. ′SN1′) cotyledons were transformed with the *Agrobacterium tumefaciens* strain LBA4404 containing the *MdoMYB121*-overexpressing constructs as described by Zhang and Blumwald et al. [59]. Three-month-old tomato plants were exposed to 300 mM NaCl solution in a growth chamber for 20 days for the salt tolerance assay. Then, the tomato plants were deprived of water for 15 days and then re-watered for 3 days for the drought tolerance assay. The tomato plants were transferred to a 4°C illuminated incubator for 13 days and grown at 22°C for an additional 3 days for the cold tolerance test.

### Apple Transformation and Stress Tolerance Assay in Transgenic Apples

Leaf explants of ‘Gala’ were transformed with the *Agrobacterium tumefaciens* strain LBA4404 containing the *MdoMYB121*-overexpressing constructs as described by Kotoda et al. [60]. The transgenic *in vitro* shoots were transferred to root-inducing medium, i.e., MS medium containing 0.1 mg/L IAA and transferred to pots containing nursery soil after one month. After six months, the self-rooted plantlets were transferred to pots to test their stress tolerance. The pot-grown plantlets were deprived of water for 15 days and re-watered for 3 days for the drought tolerance assay. The pot-grown plantlets were irrigated with 300 mM NaCl solution twice a week for 16 days for the salt tolerance assay. The apple plantlets were exposed to a temperature of 4°C for 16 days and then returned to normal conditions at 22°C for an additional 25 days for the cold tolerance test. All of the experiments were performed in triplicate.

### Measurement of Relative Water Content and Relative Electrolyte Leakage

The relative water content (RWC) under abiotic stresses was determined as indicated: RWC =  (fresh weight - dry weight)/(rehydrated weight - dry weight). The leaf cell membranes damaged were determined under abiotic stresses according to the method described by Premachandra et al. [61] and were calculated as indicated: electrolyte leakage = [1−(1−S1/S2)/(1−C1/C2)], the conductivity measurements corresponding to abiotic stresses treated leaves (S1), boiled abiotic stresses treated leaves (S2), non-abiotic stresses treated leaves (C1), and boiled non-abiotic stresses treated leaves (C2).

### Determination of Malondialdehyde (MDA) Level and Free Proline Analysis

The MDA level according to the method as described by Hodges et al. [62]. Tomato leaves were homogenized in 5 ml 10% trichloroacetic (TCA) and centrifuged at 12,000 rpm for 10 min. A volume of 2 ml of clear supernatant was added to 4 ml of 0.6% thiobarbituric acid (TBA, in 10% TCA) and incubated at 100°C in a water bath for 15 min. The reaction was cooled to room temperature, and the absorbance of the supernatant of MDA was measured at 450, 532, and 600 nm using a UV-vis spectrophotometer (UV-2450). The MDA contents (µmol g^−1^ FW) were calculated with the following formula: 6.45 (OD_532_–OD_600_)–0.56OD_450_.

The free proline content in leaves was determined by essentially following the reported methods of Bates et al. [63]. The leaves of tomato under multiple treatments were homogenized in 5 ml sulfosalicylic acid (3%), and the homogeneous mixture was centrifuged at 12,000 rpm for 15 min at 4°C. The extract was transformed into 3 ml acid ninhydrin and 2 ml glacial acid for 30 min at 100°C and cooled to 4°C in 30 min. The absorbance of the toluene layers was measured at 520 nm with a spectrophotometer (Shimadzu UV-2450, Kyoto, Japan). The proline concentration was determined using a calibration curve and expressed as µg proline g^−1^ FW.

### Na^+^ and K^+^ Contents

To measure the Na^+^ and K^+^ contents, the tomato leaves were weighed after drying for 24 h at 75°C and then digested with a 4∶1 (v:v) mixture of nitric and perchloric acids. These materials were filtered, diluted with distilled water, and subjected atomic absorption spectroscopy (Perkin Elmer AA300, CT, USA) to determine the Na^+^ and K^+^ concentrations.

## Supporting Information

Figure S1 **Phylogenetic relationships and subgroup designations in MYB proteins from apple and** ***Arabidopsis*** **.** (A) The tree represents relationships among 229 MYB proteins from apple, 132 (123 R2R3 MYBs, five R1R2R3 MYBs, and one 4R MYB) from *Arabidopsis.* The unrooted phylogenetic tree was inferred using the neighbor-joining method of the MEGA5 program. The numbers on the branches represent bootstrap values with 1000 bootstrap replicates. Bootstrap values <50% are not shown in the phylogenetic tree. The proteins are clustered into 45 subgroups based on clades at least 50% bootstrap values, which are designated with a subgroup number (e.g., S1 or H1) and marked with different colors to facilitate subgroup identification. (B) The functions of *AtMYB* genes are annotated and references are shown in Text S2. (C) Subgroups sharing one to four motifs are highlighted with blue color.
(TIF)

Figure S2 **Phylogenetic relationships of MYB proteins from apple,** ***Arabidopsis*** **, peach and pear.** In the annotation, ppa (121 members) and Pbr (167 members) MYBs are from peach and pear, respectively. The unrooted phylogenetic tree was constructed using the neighbor-joining method of the MEGA5 program with 1000 bootstrap replicates. Bootstrap values <50% are not shown in the phylogenetic tree.
(TIF)

Figure S3 **Intron-exon structures of apple** ***MYB*** **genes.** All 229 gene intron-exon structures are described on the right. Exons and introns are indicated by green boxes and single lines, respectively. The unrooted phylogenetic tree of 229 proteins from apple (on the left) was inferred using the neighbor-joining method of the MEGA5 program with 1000 bootstrap replicates.
(TIF)

Figure S4 **Expression analysis of the** ***MdoMYB121*** **gene under abiotic stress treatments.** *MdoMYB121* expression levels in response to salt, ABA, PEG and cold as revealed by qRT-PCR.
(TIF)

Figure S5 **Phylogenetic analysis of MdoMYB121 and abiotic stress-related MYBs from other species.** The tree was constructed using the neighbor-joining method of the MEGA5 program with 1000 bootstrap replicates.
(DOC)

Figure S6 **GFP-MdoMYB121 fusion proteins are subcellularly localized to the nucleus in onion epidermal cells.**
(DOC)

Table S1 ***MYB*** **genes in apple.**
(DOC)

Table S2 **Primers for RT-PCR analysis.**
(DOC)

Text S1 **Semi-quantitative RT-PCR sequencing data that do not have a corresponding EST.**
(DOC)

Text S2 **References for** ***MYB*** **genes which have been functionally characterized in** ***Arabidopsis*** **.**
(DOC)
