# Supplementary figures and images for: Genome Wide Analysis of the Apple MYB Transcription Factor Family Allows the Identification of MdoMYB121 Gene Confering Abiotic Stress Tolerance in Plants
Source: PLoS One. 2013 Jul 26;8(7):e69955. doi: 10.1371/journal.pone.0069955 (PMC3735319; doi:10.1371/journal.pone.0069955)

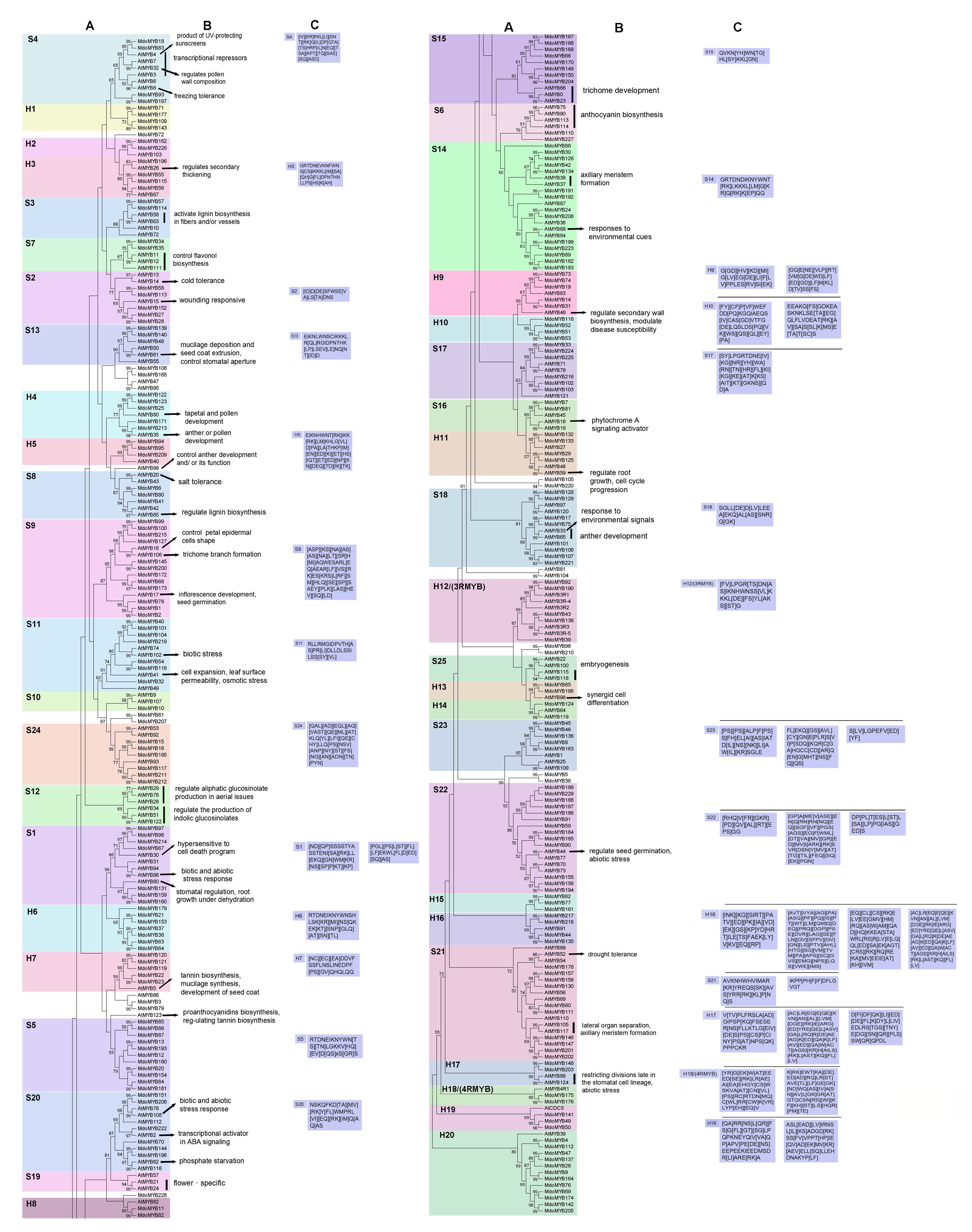

Supplement: Figure S1 — Phylogenetic relationships and subgroup designations in MYB proteins from apple and Arabidopsis . (A) The tree represents relationships among 229 MYB proteins from apple, 132 (123 R2R3 MYBs, five R1R2R3 MYBs, and one 4R MYB) from Arabidopsis. The unrooted phylogenetic tree was inferred using the neighbor-joining method of the MEGA5 program. The numbers on the branches represent bootstrap values with 1000 bootstrap replicates. Bootstrap values <50% are not shown in the phylogenetic tree. The proteins are clustered into 45 subgroups based on clades at least 50% bootstrap values, which are designated with a subgroup number (e.g., S1 or H1) and marked with different colors to facilitate subgroup identification. (B) The functions of AtMYB genes are annotated and references are shown in Text S2. (C) Subgroups sharing one to four motifs are highlighted with blue color. (TIF) [file pone.0069955.s001.tif]

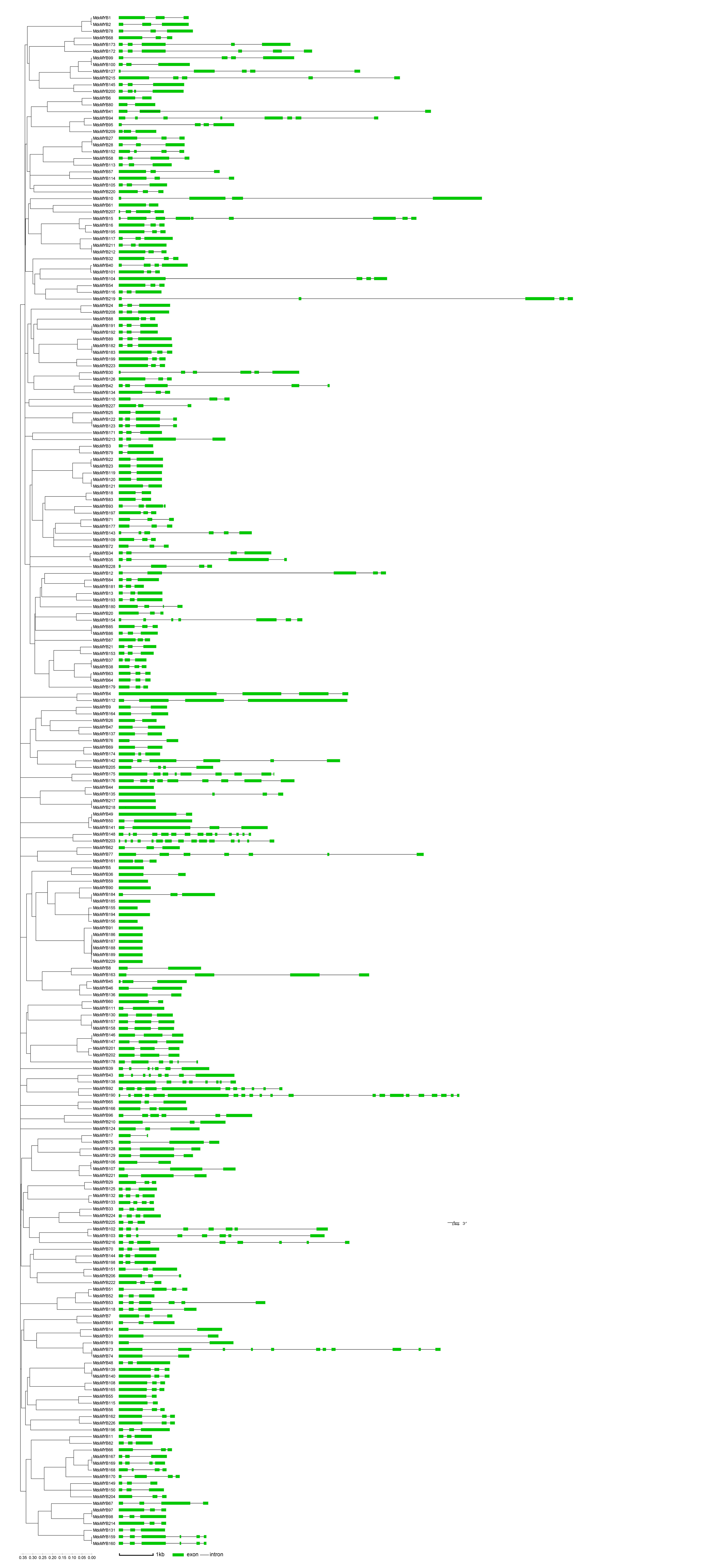

Supplement: Figure S3 — Intron-exon structures of apple MYB genes. All 229 gene intron-exon structures are described on the right. Exons and introns are indicated by green boxes and single lines, respectively. The unrooted phylogenetic tree of 229 proteins from apple (on the left) was inferred using the neighbor-joining method of the MEGA5 program with 1000 bootstrap replicates. (TIF) [file pone.0069955.s003.tif]

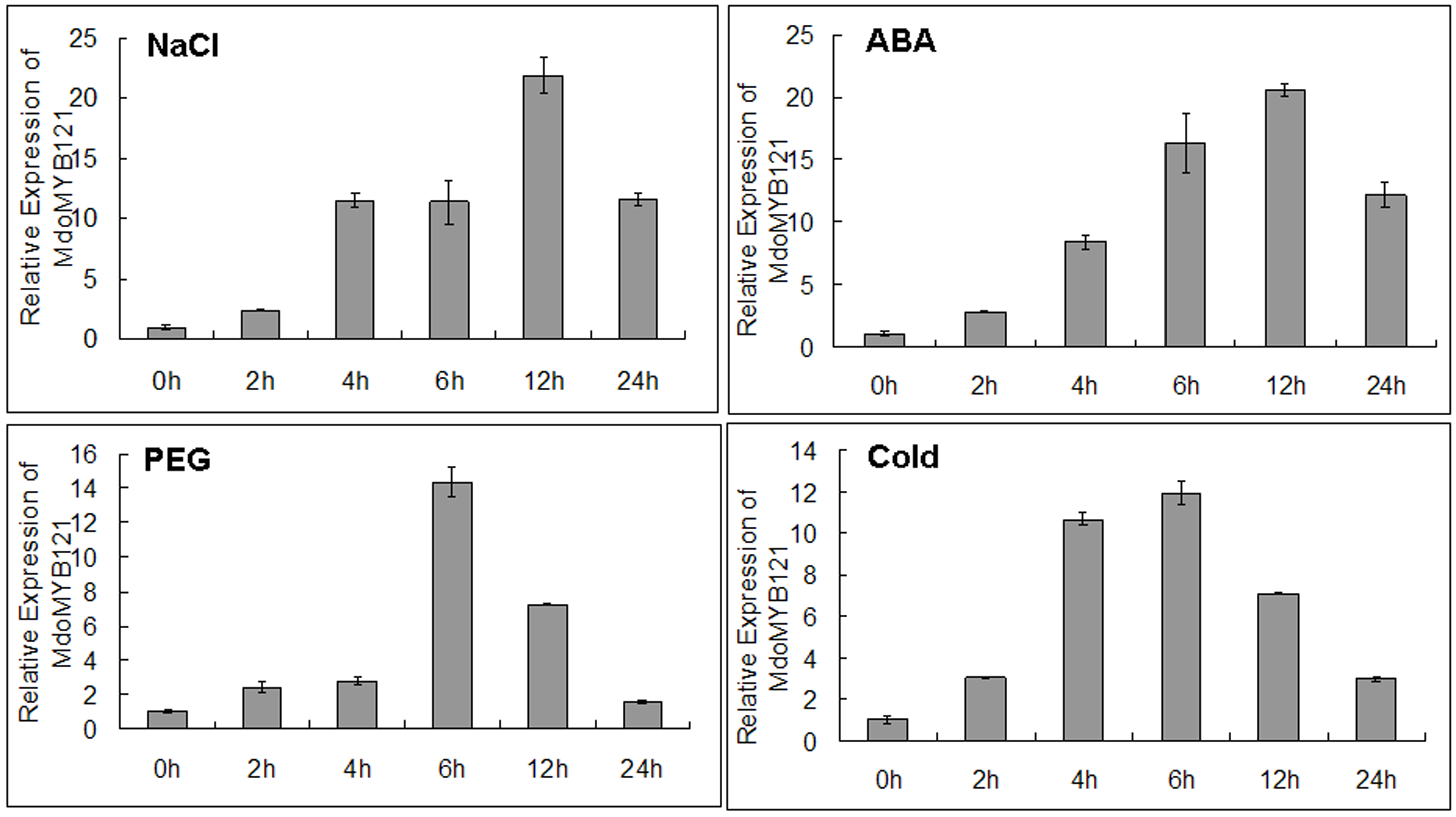

Supplement: Figure S4 — Expression analysis of the MdoMYB121 gene under abiotic stress treatments. MdoMYB121 expression levels in response to salt, ABA, PEG and cold as revealed by qRT-PCR. (TIF) [file pone.0069955.s004.tif]
