# Supplementary material for: Genome Wide Analysis of the Apple MYB Transcription Factor Family Allows the Identification of MdoMYB121 Gene Confering Abiotic Stress Tolerance in Plants
Source: PLoS One. 2013 Jul 26;8(7):e69955. doi: 10.1371/journal.pone.0069955 (PMC3735319; doi:10.1371/journal.pone.0069955)
Supplement: Figure S5 — Phylogenetic analysis of MdoMYB121 and abiotic stress-related MYBs from other species. The tree was constructed using the neighbor-joining method of the MEGA5 program with 1000 bootstrap replicates. (DOC) [file pone.0069955.s005.doc]

**Figure S5. Phylogenetic analysis of MdoMYB121 and abiotic stress-related MYBs from other species.** The tree was constructed using the neighbor-joining method of the MEGA5 program with 1000 bootstrap replicates. OsMYB, HvMYB, TaMYB, GmMYB, ZmMYB, CpMYB, and CmMYB protein from *Oryza sativa*, *Hordeum vulgare*, *Triticum aestivum*, *Glycine max*, *Zea mays*, *Carica papaya*, and *Castanea mollissima*, respectively. The full-length amino acid sequences were downloaded from the National Center for Biotechnology Information ([www.ncbi.nlm.nih.gov](http://www.ncbi.nlm.nih.gov/)).


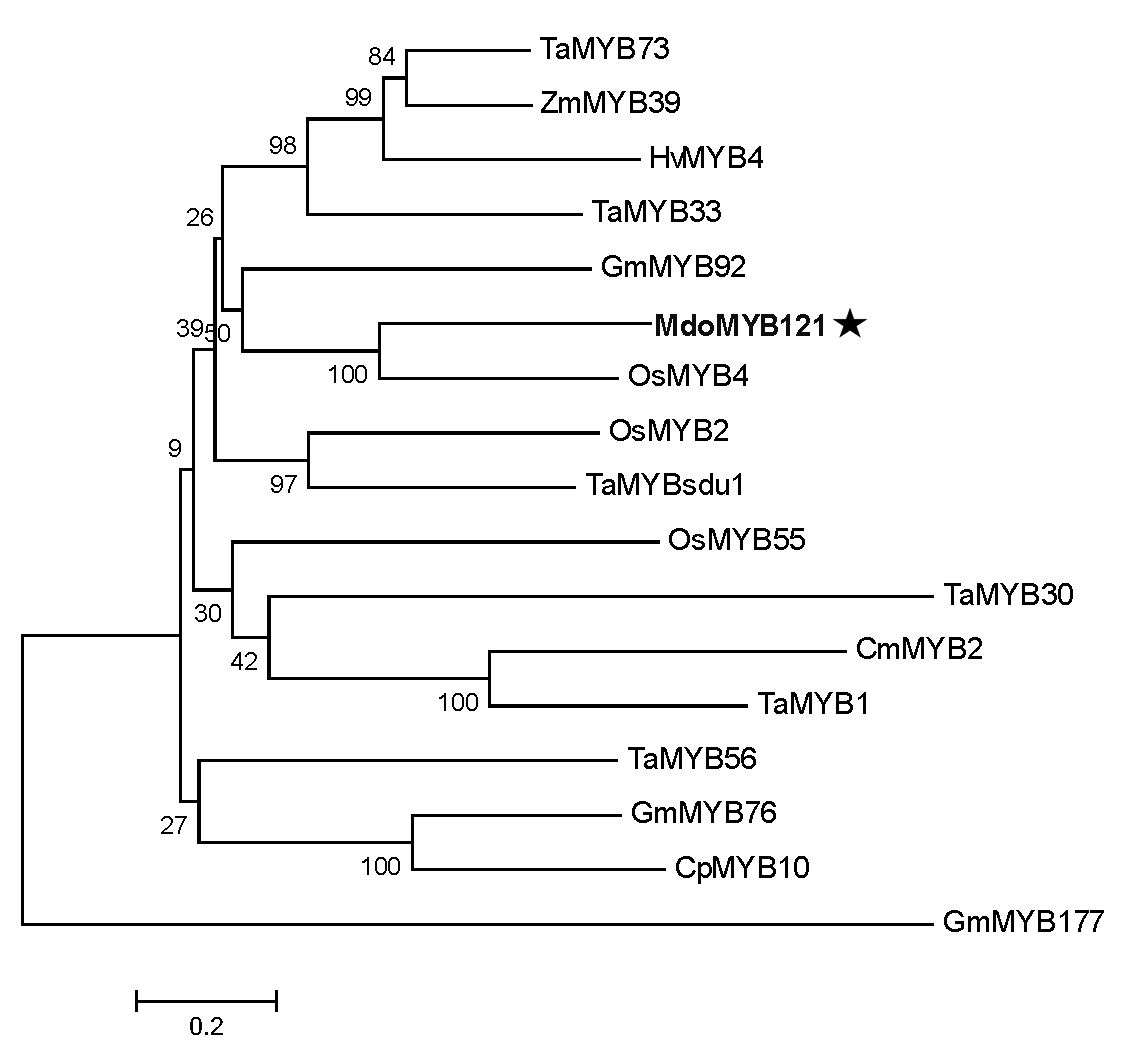


.

Reference:

He Y, Lv J, Jia Y, Wang M, Xia G (2011) [Ectopic expression of a wheat MYB transcription factor gene, TaMYB73, improves salinity stress tolerance in Arabidopsis thaliana](http://jxb.oxfordjournals.org/content/63/3/1511.short). J Exp Bot 63: 1511-1522.

Mao X, Jia D, Li A, Zhang H, Tian S, et al. (2011) Transgenic expression of a TaMYB2A confers enhanced tolerance to multiple abiotic stresses in Arabidopsis. Funct Intergr Genom 11: 445-465.

Qin Y, Wang M, Tian Y, He W, Xia G (2012) Over-expression of TaMYB33 encoding a novel wheat MYB transcription factor increases salt and drought tolerance in Arabidopsis. Mol Biol Rep 39: 7183-7192.

Liao Y, Zou H, Wang H, Zhang W, Ma Biao, et al. (2008) Soybean GmMYB76, GmMYB92, and GmMYB177 genes confer stress tolerance in transgenic Arabidopsis plants. Cell research 18: 1047-1060.

Vannini C, Locatelli F, Bracale M, Magnani E, Marsoni M, et al. (2004) Overexpression of the rice Osmyb4 gene increases chilling and freezing tolerance of Arabidopsis thaliana plants. Plant J 37: 115-127.

Rahie M, Xue G, Naghvi MR, Alizadeh H, Schenk PM (2010) A MYB gene from wheat (Triticum aestivum L.) is up-regulated during salt and drought stresses and differentially regulated between salt-tolerant and sensitive genotypes. Plant Cell Rep 29: 835-844.

Yang A, Dai X, Zhang W (2012) A R2R3-type MYB gene, OsMYB2, is involved in salt, cold, and dehydration tolerance in rice. J Exp Bot 63: 2541-2556.

Villalobos MA, Bartels D, Iturriaga G (2004) Stress tolerance and glucose insensitive phenotypes in Arabidopsis overexpressing the CpMYB10 transcription factor gene. Plant Physiol 135: 309-324.

Overexpression of a wheat MYB transcription factor gene, <i> TaMYB56-B</i>, enhances tolerances to freezing and salt stresses in transgenic Arabidopsis. Gene 505: 100-107.

El-kereamy A, Bi YM, Ranathunge K, Beatty PH, Good AG, et al. (2012) The rice R2R3-MYB transcription factor OsMYB55 is involved in the tolerance to high temperature and modulates amino acid metabolism. PlOS ONE 7: e52030.

Zhang L, Zhao G, Xia C, Jia J, Liu X, et al. (2012) A wheat R2R3-MYB gene, TaMYB30-B, improves drought stress tolerance in transgenic Arabidopsis.J Exp Bot 63: 5873-5885.

Shan H, Chen S, Jiang J, Chen F, Chen Y, et al. (2012) Heterologous expression of the chrysanthemum R2R3-MYB transcription factor CmMYB2 enhances drought and salinity tolerance, increases hypersensitivity to ABA and delays flowering in Arabidopsis thaliana. 51: 160-173.

A Myb transcription factor (TaMyb1) from wheat roots is expressed during hypoxia: roles in response to the oxygen concentration in root environment and abiotic stresses. Physiol Plantarum 129: 375-385.
