# Supplementary material for: Genome Wide Analysis of the Apple MYB Transcription Factor Family Allows the Identification of MdoMYB121 Gene Confering Abiotic Stress Tolerance in Plants
Source: PLoS One. 2013 Jul 26;8(7):e69955. doi: 10.1371/journal.pone.0069955 (PMC3735319; doi:10.1371/journal.pone.0069955)
Supplement: Figure S6 — GFP-MdoMYB121 fusion proteins are subcellularly localized to the nucleus in onion epidermal cells. (DOC) [file pone.0069955.s006.doc]

**Figure S6. GFP-MdoMYB121 fusion proteins are subcellularly localized to the nucleus in onion epidermal cells.**

To determine the subcellular localization of MdoMYB121, p35S:MdoMYB121-GFP and p35S:GFP were transiently expressed in onion (*Allium cepa*) epidermal cells. The results showed that the onion cells transformed with the p35S:GFP vector displayed fluorescence throughout the entire cells (D-E). In contrast, the green fluorescence protein (GFP) signal in the onion cell transformed with p35S:MdoMYB121-GFP was detected exclusively in the nucleus, which indicates that the *MdoMYB121* gene encodes a nucleus-localized protein (A-C).

(A, D) These photographs were taken in dark field for green fluorescence. (B, E) Bright light is used to show the morphology of the cell and (C, F) in combination. (A-C) and (D-F) for p35S:MdoMYB121-GFP and p35S:GFP plasmid control, respectively. Bars = 50 µm.

**
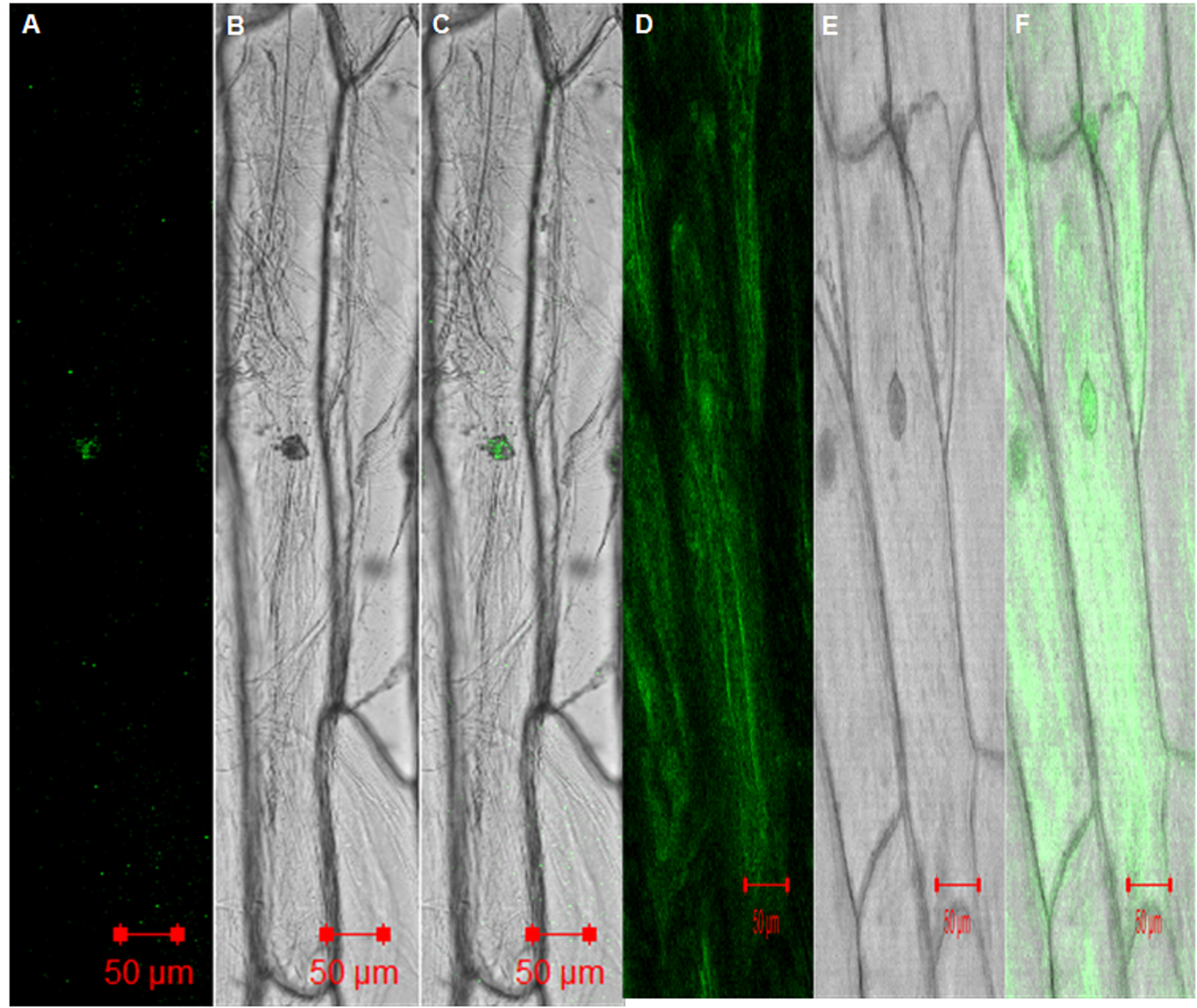
**
