# Supplementary material for: Genome Wide Analysis of the Apple MYB Transcription Factor Family Allows the Identification of MdoMYB121 Gene Confering Abiotic Stress Tolerance in Plants
Source: PLoS One. 2013 Jul 26;8(7):e69955. doi: 10.1371/journal.pone.0069955 (PMC3735319; doi:10.1371/journal.pone.0069955)
Supplement: Table S1 — MYB genes in apple. (DOC) [file pone.0069955.s007.doc]

**Table S1. *MYB* genes in apple.** Among these MdoMYBs, the predicted proteins MdoMYB39, MdoMYB43, MdoMYB92, MdoMYB138 and MdoMYB190 belong to the R1R2R3 MYB family*.* In addition, MdoMYB175 and MdoMYB176 belong to the 4R-like MYB family*.*

| ID | Gene | chromosome | ORF | protein | pI | synonym |
| --- | --- | --- | --- | --- | --- | --- |
| MDP0000308403 | *MdoMYB1* | chr1 | 1167 | 388 | 5.86 | *MdMYB14*(DQ074465) |
| MDP0000132671 | *MdoMYB2* | chr1 | 1167 | 388 | 5.79 |  |
| MDP0000157940 | *MdoMYB3* | chr1 | 864 | 287 | 8.61 |  |
| MDP0000284572 | *MdoMYB4* | chr1 | 5088 | 1695 | 5.49 |  |
| MDP0000693678 | *MdoMYB5* | chr1 | 756 | 251 | 6.01 |  |
| MDP0000787808 | *MdoMYB6* | chr1 | 756 | 251 | 5.06 | *MdMYBB*(AB370230.1) |
| MDP0000152575 | *MdoMYB7* | chr1 | 858 | 285 | 7.68 |  |
| MDP0000807341 | *MdoMYB8* | chr1 | 1239 | 412 | 6.51 | *MdMYB1*(HM122614.1) |
| MDP0000277813 | *MdoMYB9* | chr1 | 852 | 283 | 5.77 | *MdMYBR1*(HM122634.1) |
| MDP0000574942 | *MdoMYB10* | chr1 | 2868 | 955 | 8.46 |  |
| MDP0000264051 | *MdoMYB11* | chr2 | 780 | 259 | 8.79 |  |
| MDP0000592543 | *MdoMYB12* | chr2 | 1455 | 484 | 6.52 |  |
| MDP0000140609 | *MdoMYB13* | chr2 | 987 | 328 | 6.3 |  |
| MDP0000204495 | *MdoMYB14* | chr2 | 1032 | 343 | 5.11 | *MdMYB20*(DQ074468.1) |
| MDP0000302984 | *MdoMYB15* | chr2 | 2385 | 794 | 6.28 |  |
| MDP0000228252 | *MdoMYB16* | chr2 | 1026 | 341 | 5.58 |  |
| MDP0000841703 | *MdoMYB17* | chr2 | 381 | 126 | 8.95 |  |
| MDP0000950559 | *MdoMYB18* | chr2 | 768 | 255 | 8.79 | *MdMYB16*(HM122617.1) |
| MDP0000736994 | *MdoMYB19* | chr2 | 1014 | 337 | 5.83 |  |
| MDP0000318013 | *MdoMYB20* | chr3 | 807 | 268 | 6.12 |  |
| MDP0000931057 | *MdoMYB21* | chr3 | 678 | 225 | 9.6 |  |
| MDP0000125457 | *MdoMYB22* | chr3 | 1125 | 374 | 8.16 | *MdMYB12*(HM122616.1) |
| MDP0000791870 | *MdoMYB23* | chr3 | 1125 | 374 | 6.7 | *MdMYB12*(HM122626.1) |
| MDP0000851495 | *MdoMYB24* | chr3 | 1161 | 386 | 6.89 |  |
| MDP0000896551 | *MdoMYB25* | chr3 | 1083 | 360 | 8.03 |  |
| MDP0000209471 | *MdoMYB26* | chr3 | 912 | 303 | 9.41 |  |
| MDP0000435315 | *MdoMYB27* | chr3 | 813 | 270 | 5.25 | *MdMYB23*(DQ074471.1) |
| MDP0000230141 | *MdoMYB28* | chr3 | 813 | 270 | 5.25 | *MdMYB23*(DQ074471.1) |
| MDP0000143156 | *MdoMYB29* | chr3 | 774 | 257 | 8.12 |  |
| MDP0000229498 | *MdoMYB30* | chr3 | 1494 | 497 | 8.37 |  |
| MDP0000722954 | *MdoMYB31* | chr3 | 1044 | 347 | 5.03 |  |
| MDP0000144751 | *MdoMYB32* | chr3 | 1032 | 343 | 7.17 |  |
| MDP0000183451 | *MdoMYB33* | chr3 | 783 | 260 | 8.99 |  |
| MDP0000119725 | *MdoMYB34* | chr3 | 1212 | 403 | 4.61 |  |
| MDP0000607330 | *MdoMYB35* | chr3 | 1503 | 500 | 5.41 |  |
| MDP0000298689 | *MdoMYB36* | chr4 | 960 | 319 | 7.21 |  |
| MDP0000253904 | *MdoMYB37* | chr4 | 594 | 197 | 9.26 |  |
| MDP0000226215 | *MdoMYB38* | chr4 | 594 | 197 | 9.17 |  |
| MDP0000181570 | *MdoMYB39* | chr4 | 1308 | 435 | 8.52 |  |
| MDP0000623034 | *MdoMYB40* | chr4 | 1149 | 382 | 7.59 |  |
| MDP0000682032 | *MdoMYB41* | chr4 | 1005 | 334 | 5.22 |  |
| MDP0000245436 | *MdoMYB42* | chr4 | 1185 | 394 | 8.83 |  |
| MDP0000197330 | *MdoMYB43* | chr4 | 1779 | 592 | 8.06 |  |
| MDP0000708334 | *MdoMYB44* | chr4 | 1062 | 353 | 9.38 |  |
| MDP0000319124 | *MdoMYB45* | chr4 | 1251 | 416 | 5.64 |  |
| MDP0000203733 | *MdoMYB46* | chr4 | 1176 | 391 | 5.37 |  |
| MDP0000221993 | *MdoMYB47* | chr4 | 924 | 307 | 6.09 |  |
| MDP0000653903 | *MdoMYB48* | chr4 | 1245 | 414 | 8.52 |  |
| MDP0000191083 | *MdoMYB49* | chr4 | 1899 | 632 | 8.75 |  |
| MDP0000151195 | *MdoMYB50* | chr4 | 1899 | 632 | 8.75 |  |
| MDP0000245462 | *MdoMYB51* | chr5 | 849 | 282 | 7.07 |  |
| MDP0000885666 | *MdoMYB52* | chr5 | 720 | 239 | 7.64 |  |
| MDP0000289283 | *MdoMYB53* | chr5 | 1182 | 393 | 7.53 |  |
| MDP0000197283 | *MdoMYB54* | chr5 | 1059 | 352 | 6 | *MdMYB36*(HM122628.1) |
| MDP0000289671 | *MdoMYB55* | chr5 | 966 | 321 | 5.66 |  |
| MDP0000157506 | *MdoMYB56* | chr5 | 987 | 328 | 6.46 |  |
| MDP0000133542 | *MdoMYB57* | chr5 | 1092 | 363 | 5.26 |  |
| MDP0000145050 | *MdoMYB58* | chr5 | 903 | 300 | 5.87 |  |
| MDP0000187872 | *MdoMYB59* | chr5 | 885 | 294 | 7.66 |  |
| MDP0000852158 | *MdoMYB60* | chr5 | 1056 | 351 | 9.41 |  |
| MDP0000286526 | *MdoMYB61* | chr6 | 1077 | 358 | 6.21 |  |
| MDP0000237596 | *MdoMYB62* | chr6 | 915 | 304 | 5.85 |  |
| MDP0000133817 | *MdoMYB63* | chr6 | 624 | 207 | 6.85 |  |
| MDP0000143276 | *MdoMYB64* | chr6 | 624 | 207 | 6.85 |  |
| MDP0000215359 | *MdoMYB65* | chr6 | 1458 | 485 | 6.5 |  |
| MDP0000578193 | *MdoMYB66* | chr6 | 687 | 228 | 7.01 |  |
| MDP0000149535 | *MdoMYB67* | chr6 | 1257 | 418 | 6.7 | *MdMYB31*(HM122623.1) |
| MDP0000868578 | *MdoMYB68* | chr6 | 972 | 323 | 7.74 |  |
| MDP0000241185 | *MdoMYB69* | chr6 | 921 | 306 | 8.58 | *MdMYBR*(HM122641.1) |
| MDP0000167107 | *MdoMYB70* | chr6 | 879 | 292 | 6.25 | *MdMYB8*(DQ267899.1) |
| MDP0000209761 | *MdoMYB71* | chr6 | 600 | 199 | 9.6 |  |
| MDP0000872821 | *MdoMYB72* | chr6 | 561 | 186 | 9.28 |  |
| MDP0000296109 | *MdoMYB73* | chr7 | 2004 | 667 | 8.21 |  |
| MDP0000167639 | *MdoMYB74* | chr7 | 1026 | 341 | 5.59 |  |
| MDP0000147309 | *MdoMYB75* | chr7 | 1656 | 551 | 4.99 |  |
| MDP0000610242 | *MdoMYB76* | chr7 | 660 | 219 | 6.72 |  |
| MDP0000319634 | *MdoMYB77* | chr7 | 1455 | 484 | 7.49 |  |
| MDP0000127521 | *MdoMYB78* | chr7 | 1008 | 335 | 6.09 |  |
| MDP0000915330 | *MdoMYB79* | chr7 | 888 | 295 | 9.16 |  |
| MDP0000602739 | *MdoMYB80* | chr7 | 840 | 279 | 4.92 |  |
| MDP0000896053 | *MdoMYB81* | chr7 | 891 | 296 | 6.65 |  |
| MDP0000167314 | *MdoMYB82* | chr8 | 774 | 257 | 9.15 |  |
| MDP0000249611 | *MdoMYB83* | chr8 | 768 | 255 | 8.94 |  |
| MDP0000210851 | *MdoMYB84* | chr8 | 873 | 290 | 5.9 | *MdMYB9*(DQ267900.1) |
| MDP0000166645 | *MdoMYB85* | chr8 | 744 | 247 | 5.37 |  |
| MDP0000209939 | *MdoMYB86* | chr8 | 744 | 247 | 5.37 |  |
| MDP0000287232 | *MdoMYB87* | chr8 | 771 | 256 | 5.67 |  |
| MDP0000266156 | *MdoMYB88* | chr8 | 894 | 297 | 6.52 |  |
| MDP0000786507 | *MdoMYB89* | chr8 | 1254 | 417 | 6.06 |  |
| MDP0000463846 | *MdoMYB90* | chr8 | 960 | 319 | 8.67 |  |
| MDP0000894463 | *MdoMYB91* | chr8 | 729 | 242 | 9.18 |  |
| MDP0000179225 | *MdoMYB92* | chr8 | 3063 | 1020 | 5.23 |  |
| MDP0000135594 | *MdoMYB93* | chr8 | 804 | 267 | 9.31 |  |
| MDP0000303239 | *MdoMYB94* | chr9 | 1341 | 446 | 7.57 |  |
| MDP0000241111 | *MdoMYB95* | chr9 | 981 | 326 | 5.44 |  |
| MDP0000284846 | *MdoMYB96* | chr9 | 1572 | 523 | 8.58 |  |
| MDP0000219359 | *MdoMYB97* | chr9 | 1029 | 342 | 8.74 | *MdMYB30*(HM122622.1) |
| MDP0000149102 | *MdoMYB98* | chr9 | 1029 | 342 | 8.74 | *MdMYB30*(HM122622.1) |
| MDP0000278681 | *MdoMYB99* | chr9 | 1323 | 440 | 6.63 |  |
| MDP0000121476 | *MdoMYB100* | chr9 | 1170 | 389 | 6.35 |  |
| MDP0000228930 | *MdoMYB101* | chr9 | 996 | 331 | 8.27 |  |
| MDP0000250031 | *MdoMYB102* | chr9 | 1185 | 394 | 9.9 |  |
| MDP0000305363 | *MdoMYB103* | chr9 | 1281 | 426 | 9.8 |  |
| MDP0000710259 | *MdoMYB104* | chr9 | 2055 | 684 | 8.29 |  |
| MDP0000155426 | *MdoMYB105* | chr9 | 762 | 253 | 7.59 | *MdMYB25*(HM122620.1) |
| MDP0000176798 | *MdoMYB106* | chr9 | 924 | 307 | 9.83 |  |
| MDP0000151688 | *MdoMYB107* | chr9 | 1485 | 494 | 5.93 |  |
| MDP0000256645 | *MdoMYB108* | chr9 | 1125 | 374 | 5.69 |  |
| MDP0000159011 | *MdoMYB109* | chr9 | 720 | 239 | 8.87 | *MdMYB111*(HM122615.1) |
| MDP0000573302 | *MdoMYB110* | chr9 | 708 | 235 | 8.91 |  |
| MDP0000291518 | *MdoMYB111* | chr10 | 1071 | 356 | 9.25 |  |
| MDP0000668138 | *MdoMYB112* | chr10 | 5133 | 1710 | 5.83 |  |
| MDP0000897594 | *MdoMYB113* | chr10 | 804 | 267 | 5.12 |  |
| MDP0000215675 | *MdoMYB114* | chr10 | 1098 | 365 | 4.87 |  |
| MDP0000226667 | *MdoMYB115* | chr10 | 1002 | 333 | 5.02 |  |
| MDP0000492221 | *MdoMYB116* | chr10 | 996 | 331 | 6.35 |  |
| MDP0000145757 | *MdoMYB117* | chr10 | 1086 | 361 | 5.72 |  |
| MDP0000131822 | *MdoMYB118* | chr10 | 1047 | 348 | 9.08 |  |
| MDP0000723614 | *MdoMYB119* | chr11 | 1104 | 367 | 9.57 |  |
| MDP0000655330 | *MdoMYB120* | chr11 | 1098 | 365 | 8.31 |  |
| MDP0000196982 | *MdoMYB121* | chr11 | 1095 | 364 | 8.31 |  |
| MDP0000420216 | *MdoMYB122* | chr11 | 1041 | 346 | 6.6 |  |
| MDP0000701148 | *MdoMYB123* | chr11 | 1041 | 346 | 6.6 |  |
| MDP0000555913 | *MdoMYB124* | chr11 | 1494 | 497 | 6.26 |  |
| MDP0000177010 | *MdoMYB125* | chr11 | 774 | 257 | 8.47 |  |
| MDP0000124049 | *MdoMYB126* | chr11 | 1062 | 353 | 6.51 |  |
| MDP0000274301 | *MdoMYB127* | chr11 | 1086 | 361 | 9.39 |  |
| MDP0000308617 | *MdoMYB128* | chr11 | 1635 | 544 | 6.88 |  |
| MDP0000179306 | *MdoMYB129* | chr11 | 1635 | 544 | 6.88 |  |
| MDP0000755899 | *MdoMYB130* | chr12 | 1206 | 401 | 8.25 |  |
| MDP0000805075 | *MdoMYB131* | chr12 | 978 | 325 | 6.37 |  |
| MDP0000221801 | *MdoMYB132* | chr12 | 705 | 234 | 5.12 |  |
| MDP0000819856 | *MdoMYB133* | chr12 | 690 | 229 | 5.46 |  |
| MDP0000587648 | *MdoMYB134* | chr12 | 969 | 322 | 8.6 |  |
| MDP0000282792 | *MdoMYB135* | chr12 | 1407 | 468 | 9.33 |  |
| MDP0000478512 | *MdoMYB136* | chr12 | 1170 | 389 | 5.52 | *MdMYB35*(HM122627.1) |
| MDP0000142580 | *MdoMYB137* | chr12 | 915 | 304 | 6.8 |  |
| MDP0000219581 | *MdoMYB138* | chr12 | 1743 | 580 | 8.43 |  |
| MDP0000932804 | *MdoMYB139* | chr12 | 1236 | 411 | 7.71 |  |
| MDP0000601420 | *MdoMYB140* | chr12 | 1233 | 410 | 7.71 |  |
| MDP0000878701 | *MdoMYB141* | chr12 | 2937 | 978 | 5.24 |  |
| MDP0000248333 | *MdoMYB142* | chr13 | 2295 | 764 | 7.64 |  |
| MDP0000250597 | *MdoMYB143* | chr13 | 906 | 301 | 9.24 |  |
| MDP0000934638 | *MdoMYB144* | chr13 | 891 | 296 | 6.06 |  |
| MDP0000716457 | *MdoMYB145* | chr13 | 1161 | 386 | 6.6 |  |
| MDP0000136541 | *MdoMYB146* | chr13 | 1359 | 452 | 8.1 | *MdMYB32*(HM122624.1) |
| MDP0000146675 | *MdoMYB147* | chr13 | 1359 | 452 | 8.1 |  |
| MDP0000210970 | *MdoMYB148* | chr13 | 1572 | 523 | 5.74 |  |
| MDP0000199965 | *MdoMYB149* | chr13 | 447 | 148 | 10.01 |  |
| MDP0000537324 | *MdoMYB150* | chr13 | 678 | 225 | 6.72 |  |
| MDP0000274300 | *MdoMYB151* | chr13 | 1056 | 351 | 6.04 |  |
| MDP0000168728 | *MdoMYB152* | chr13 | 666 | 221 | 4.66 |  |
| MDP0000124555 | *MdoMYB153* | chr13 | 672 | 223 | 9.51 |  |
| MDP0000437717 | *MdoMYB154* | chr13 | 1125 | 374 | 9.23 |  |
| MDP0000232454 | *MdoMYB155* | chr13 | 573 | 190 | 9.41 |  |
| MDP0000196986 | *MdoMYB156* | chr13 | 573 | 190 | 9.41 |  |
| MDP0000172476 | *MdoMYB157* | chr14 | 1221 | 406 | 8.91 |  |
| MDP0000878006 | *MdoMYB158* | chr14 | 1221 | 406 | 8.91 |  |
| MDP0000163673 | *MdoMYB159* | chr14 | 1161 | 386 | 6.9 |  |
| MDP0000168550 | *MdoMYB160* | chr14 | 1161 | 386 | 7.2 |  |
| MDP0000552725 | *MdoMYB161* | chr14 | 864 | 287 | 6.02 |  |
| MDP0000318376 | *MdoMYB162* | chr14 | 972 | 323 | 7.12 |  |
| MDP0000284922 | *MdoMYB163* | chr14 | 1959 | 652 | 5.97 |  |
| MDP0000809640 | *MdoMYB164* | chr14 | 861 | 286 | 5.75 |  |
| MDP0000852549 | *MdoMYB165* | chr14 | 1113 | 370 | 5.52 |  |
| MDP0000263562 | *MdoMYB166* | chr14 | 1680 | 559 | 7.93 |  |
| MDP0000642761 | *MdoMYB167* | chr14 | 705 | 234 | 6.45 |  |
| MDP0000266683 | *MdoMYB168* | chr14 | 576 | 191 | 4.93 |  |
| MDP0000193637 | *MdoMYB169* | chr14 | 618 | 205 | 5.33 |  |
| MDP0000650225 | *MdoMYB170* | chr14 | 687 | 228 | 9.61 |  |
| MDP0000697636 | *MdoMYB171* | chr14 | 900 | 299 | 6.9 |  |
| MDP0000136874 | *MdoMYB172* | chr14 | 1530 | 509 | 8.79 |  |
| MDP0000298505 | *MdoMYB173* | chr14 | 1854 | 617 | 8.9 |  |
| MDP0000853568 | *MdoMYB174* | chr14 | 999 | 332 | 7.41 |  |
| MDP0000234846 | *MdoMYB175* | chr14 | 2244 | 747 | 9.05 |  |
| MDP0000184989 | *MdoMYB176* | chr14 | 2535 | 844 | 6.48 |  |
| MDP0000211677 | *MdoMYB177* | chr14 | 591 | 196 | 9.28 | *MdMYB17*(HM122618.1) |
| MDP0000265114 | *MdoMYB178* | chr14 | 984 | 327 | 8.49 |  |
| MDP0000261265 | *MdoMYB179* | chr15 | 600 | 199 | 9.59 |  |
| MDP0000184538 | *MdoMYB180* | chr15 | 840 | 279 | 9.02 |  |
| MDP0000031172 | *MdoMYB181* | chr15 | 528 | 175 | 10.29 |  |
| MDP0000124992 | *MdoMYB182* | chr15 | 1254 | 417 | 6.89 |  |
| MDP0000262514 | *MdoMYB183* | chr15 | 1254 | 417 | 6.89 |  |
| MDP0000275800 | *MdoMYB184* | chr15 | 1290 | 429 | 9.44 |  |
| MDP0000165715 | *MdoMYB185* | chr15 | 954 | 317 | 7.68 | *MdMYB34*(HM122626.1) |
| MDP0000480596 | *MdoMYB186* | chr15 | 717 | 238 | 9.18 |  |
| MDP0000784297 | *MdoMYB187* | chr15 | 717 | 238 | 9.18 |  |
| MDP0000477900 | *MdoMYB188* | chr15 | 717 | 238 | 9.18 |  |
| MDP0000823270 | *MdoMYB189* | chr15 | 717 | 238 | 9.18 |  |
| MDP0000295807 | *MdoMYB190* | chr15 | 4455 | 1484 | 5.08 |  |
| MDP0000836365 | *MdoMYB191* | chr15 | 771 | 256 | 5.62 |  |
| MDP0000785820 | *MdoMYB192* | chr15 | 771 | 256 | 5.62 |  |
| MDP0000887107 | *MdoMYB193* | chr15 | 984 | 327 | 6.57 |  |
| MDP0000629440 | *MdoMYB194* | chr15 | 939 | 312 | 7.69 | *MdMYB6*(DQ074461) |
| MDP0000320772 | *MdoMYB195* | chr15 | 1038 | 345 | 5.59 |  |
| MDP0000148894 | *MdoMYB196* | chr15 | 1122 | 373 | 5.94 |  |
| MDP0000175918 | *MdoMYB197* | chr15 | 933 | 310 | 9.14 | *MdMYB3*(JN544704.1) |
| MDP0000659260 | *MdoMYB198* | chr16 | 897 | 298 | 5.83 | *MdMYB7*(DQ074462.1) |
| MDP0000375685 | *MdoMYB199* | chr16 | 1176 | 391 | 7.15 |  |
| MDP0000204699 | *MdoMYB200* | chr16 | 1221 | 406 | 6.68 |  |
| MDP0000232291 | *MdoMYB201* | chr16 | 1215 | 404 | 8.67 |  |
| MDP0000181178 | *MdoMYB202* | chr16 | 1356 | 451 | 8.45 |  |
| MDP0000165523 | *MdoMYB203* | chr16 | 1620 | 539 | 6.26 |  |
| MDP0000164048 | *MdoMYB204* | chr16 | 654 | 217 | 8.97 |  |
| MDP0000208448 | *MdoMYB205* | chr16 | 1008 | 335 | 7.28 |  |
| MDP0000812154 | *MdoMYB206* | chr16 | 891 | 296 | 6.7 |  |
| MDP0000910638 | *MdoMYB207* | chr16 | 858 | 285 | 10.07 |  |
| MDP0000809452 | *MdoMYB208* | chr16 | 1161 | 386 | 6.05 |  |
| MDP0000200600 | *MdoMYB209* | chr17 | 924 | 307 | 5.38 |  |
| MDP0000199939 | *MdoMYB210* | chr17 | 1590 | 529 | 6.62 |  |
| MDP0000126343 | *MdoMYB211* | chr17 | 1062 | 353 | 5.78 |  |
| MDP0000123873 | *MdoMYB212* | chr17 | 1062 | 353 | 5.78 |  |
| MDP0000258046 | *MdoMYB213* | chr17 | 1410 | 469 | 7.61 |  |
| MDP0000144744 | *MdoMYB214* | chr17 | 1029 | 342 | 6.8 |  |
| MDP0000742771 | *MdoMYB215* | chr17 | 1446 | 481 | 6.06 |  |
| MDP0000286143 | *MdoMYB216* | chr17 | 1215 | 404 | 8.93 |  |
| MDP0000252887 | *MdoMYB217* | chr17 | 1113 | 370 | 9.39 |  |
| MDP0000422537 | *MdoMYB218* | chr17 | 1101 | 366 | 9.39 |  |
| MDP0000468201 | *MdoMYB219* | chr17 | 1266 | 421 | 7.63 |  |
| MDP0000148689 | *MdoMYB220* | chr17 | 840 | 279 | 5.82 |  |
| MDP0000175835 | *MdoMYB221* | chr17 | 1581 | 526 | 5.66 |  |
| MDP0000823458 | *MdoMYB222* | chr17 | 873 | 290 | 4.91 |  |
| MDP0000322479 | *MdoMYB223* | chr0 | 1140 | 379 | 5.81 |  |
| MDP0000133412 | *MdoMYB224* | chr0 | 867 | 288 | 8.89 |  |
| MDP0000133416 | *MdoMYB225* | chr0 | 489 | 162 | 9.87 |  |
| MDP0000263595 | *MdoMYB226* | chr0 | 984 | 327 | 8.02 |  |
| MDP0000192051 | *MdoMYB227* | chr0 | 756 | 251 | 8.55 |  |
| MDP0000317209 | *MdoMYB228* | chr0 | 768 | 255 | 7.71 |  |
| MDP0000641708 | *MdoMYB229* | chr0 | 717 | 238 | 9.18 |  |
