# Supplementary material for: Genome Wide Analysis of the Apple MYB Transcription Factor Family Allows the Identification of MdoMYB121 Gene Confering Abiotic Stress Tolerance in Plants
Source: PLoS One. 2013 Jul 26;8(7):e69955. doi: 10.1371/journal.pone.0069955 (PMC3735319; doi:10.1371/journal.pone.0069955)
Supplement: Table S2 — Primers for RT-PCR analysis. (DOC) [file pone.0069955.s008.doc]

**Table S2.** Primers for RT-PCRs analysis.

| *MdoMYB11* | Forward:5'-GCTAGCAGGTCGACTCACAG-3' |
| --- | --- |
|  | Reverse:5'-GTTGCCCAGTTGCCTTCACC-3' |
| *MdoMYB22* | Forward:5'-TCTCCATTTGTCTTCGCATTCG-3' |
|  | Reverse:5'-CTTCAAACCCACCTTTACGCAAC-3' |
| *MdoMYB54* | Forward:5'-GTACATGAACAGTTGAGCACAC-3' |
|  | Reverse:5'-CAGCTCTTCCCGCATCTTTGTA-3' |
| *MdoMYB67* | Forward:5'-CGCAACTGCCTGCAATCTTCT-3' |
|  | Reverse:5'-CCGGGCGGAGATAATTAGTCC-3' |
| *MdoMYB97* | Forward:5'-GGAGACATTCTGTGGTGGTGAT-3' |
|  | Reverse:5'-GCCATTCCAAGCAAAATCCAAT-3' |
| *MdoMYB107* | Forward:5'-:AGGTGGCCGATAACTTCACAT-3' |
|  | Reverse:5'-CTACCAAAATCGCGTCCTCTGC-3' |
| *MdoMYB109* | Forward:5'-CATGTTCTGCCATTACCTGGGC-3' |
|  | Reverse:5'-GCACGACGGTTGTTCAAATG-3' |
| *MdoMYB121* | Forward:5'-TCATCCCCCATCCTCACTACCA-3' |
|  | Reverse:5'-TCGTTTGCATCTTAGCACTTGC-3' |
| *MdoMYB133* | Forward:5'-AGCCAGTGGTGGAGTTCCCA-3' |
|  | Reverse:5'-TCCCACCTCCGATTCCCCTTA-3' |
| *MdoMYB136* | Forward:5'-GATAGAATGGAAACGGAAGTCGC-3' |
|  | Reverse:5'-GATTCCTCGGGCGATCAAGCT-3' |
| *MdoMYB146* | Forward:5'-TCCTACCTAGCTTTCACTAGCG-3' |
|  | Reverse:5'-GAGAAAGAGAGCCCATATCGGC-3' |
| *MdoMYB148* | Forward:5'-GGGATCATCTGGGAGCGTC-3' |
|  | Reverse:5'-CCCAATTTTCTGTTCCATGGGTG-3' |
| *MdoMYB155* | Forward:5'-AACCAGCAGCTCTCGACTTT-3' |
|  | Reverse:5'-ATCGACTTGCTGATCAGCGAC-3' |
| *MdoMYB185* | Forward:5'-GCCAACCCCCTCTTTCTCCTT-3' |
|  | Reverse:5'-AAGACTTGCCGGATCGACCA-3' |
| *MdoMYB197* | Forward:5'-TGATCCAGAAGAAGAAACAGATGG-3' |
|  | Reverse:5'-GCGGAGGTAGTTTATCCATCTC-3' |
| *MdoMYB199* | Forward:5'-GAAACCAGGCTCATCACAACA-3' |
|  | Reverse:5'-TGCCGTGCTTCTCGATGTAAG-3' |
| *MdoMYB206* | Forward:5'-GGACAGTTGAGGAGACCTAGC-3' |
|  | Reverse:5'-CAGTCCTTCCTGGCAAGTGTT-3' |
| *MdoMYB222* | Forward:5'-TTGTCTCCCAGCTCAAACCAGA-3' |
|  | Reverse:5'-CCAAGGAATTCCAGCGACCT-3' |
