# Supplementary material for: Genome Wide Analysis of the Apple MYB Transcription Factor Family Allows the Identification of MdoMYB121 Gene Confering Abiotic Stress Tolerance in Plants
Source: PLoS One. 2013 Jul 26;8(7):e69955. doi: 10.1371/journal.pone.0069955 (PMC3735319; doi:10.1371/journal.pone.0069955)
Supplement: Text S1 — Semi-quantitative RT-PCR sequencing data that do not have a corresponding EST. (DOC) [file pone.0069955.s009.doc]

**Text S1. Semi-quantitative** **RT-PCR sequencing data that do not have a corresponding EST.**

To verify that these genes really exist in the apple genome, blasts were conducted through the GenBank database using the DNA sequences of 18 *MdoMYB* gene models. The results showed that cDNA ESTs HM122616.1, HM122628.1, HM122623.1, HM122622.1, HM122615.1, HM122627, HM122624.1, HM122626.1, and JN544704.1 correspond to *MdoMYB22*, *54*, *67*, *97*, *109*, *136*, *146*, *185*, and *197*, respectively, which indicates that these *MdMYB* genes really exist in the apple genome. To examine whether the *MdoMYB11*, *107*, *133*, *121*, *148*, *155*, *199*, *206* and *222* genes are really expressed in apple, their cDNA fragments were amplified for sequencing. The results showed that all of these genes produced cDNA as predicted, which indicates that they do exist and are expressed in the apple genome. The primers are specific for gene models and listed in Table S3.

*MdoMYB11*

GCTAGCAGGTCGACTCACAGAAAACAAAAATATCGATCATTTTTGGATGACCGGAAACAAAAGATCATCATCAGTTGTGAAAGCAGGAAATGCACAGCCGAAGAAGAACTTGTGGAAGCCAGAGGAGGACTTGATTCTCCGACAATATGTGGAAACTCATGGTGAAGGCAACTGGGCAAC

*MdoMYB107*

AGGTGGCCGATAACTTCACATATAACTAATAGAGAGCTAGGTGGGGTGTAGGCGTAAAAGCCTTCATGATAATCAACAACAGCGGAGTTATGGACAGAGTTCATGAGACGGCGGTAGGAGCTCAGAGAGGCGGCTCCCACCACCACCAATCACCAGCGGGGCCTCAAGAAGGGGCCCTGGACGGGAGCAGAGGACGCGATTTTGGTAG

*MdoMYB121*

TCATCCCCCATCCTCACTACCAAACAAATTAAAGATGCAAAACTAAAATCTACCTACACACAAACGCAGCATCTTAAAACCTCACAGAAGTAAGAAAAACAAGATCAGGTTCTCAAGATATCAAACCTCCATTTGTATTCGCATTCGATCCACTCCTACTCAGAAGAAAAAAAAAMACTGAAAAAGCTTTACAATGAGGAACCCATCGTCTTCGTCGAAAGCAGCRGCAGCAGCAAGTGCTAAGATGCAAACGA

*MdoMYB133*

AGCCAGTGGTGGAGTTCCCACAAACTAGTGGAGTTTATGTAAGAGGTCTCATTTTTYCCTTTTCCCCAACTCTTTTGCCACCYGGTTCTGTATATAAGGGTTTAGCTTCTCTTGGTTTGTCACACCAATGATGACTTGTCAAGCAGCCATGGATGGAGAGAAACTGCGTAAAGGTCCGGGCTCGAAGAGGAAGATAACATCCAC

*MdoMYB148*

GGGATCATCTGGGAGCGTCAGAGAGAGACAGAGAGAGACAGAGAGAGAGTGAGAGTGAGGAGTGAGAAACTGTGAGATAGCTAGAGAGAGAGGTGAAATTAAAAACCCAAATGCCGCAGGAGGAGTCAAAGAAGAAGGAGCGCCACATCGTTAGTTGGTCTCAAGAGGAAGATGATATACTGAGGAATCAAATTAACACCCATGGAACAGAAAATTGGG

*MdoMYB155*

AACCAGCAGCTCTCGACTTTCAAAATTCAAAAATTTACAGAGATTAAAATCGAAAAAAAATAAAATTAAAGAGAGAGGTCGTGTGAGTTAATTGCGATGGCGGTGATCAGGAAGGATATGGATCGAATCAAGGGTCCATGGAGCCCCGAGGAGGACGACTCGCTCCAGAAGCTGGTGCAGAAGCACGGCCCCAGGAACTGGTCGCTGATCAGCAAGTCGATTCCTGGTCGGTCCGGGAAGTCGTGCCGGCTGCGGTGGTGCAA

*MdoMYB199*

GAAACCAGGCTCATCACAACATAACCCACACAATAAGAAAAAAAGCCAAGTTAAAGTAAAAGCCATGGGGAGAGCTCCTTGCTGTGACAAAGCCAACGTCAAGAAAGGCCCTTGGTCACCAGAAGAAGATGCCACCCTCAAGGCTTACATCGAGAAGCACGGCAA

*MdoMYB206*

GGACAGTTGAGGAGACCTAGCTCTCATGAATTACATTGCTAACCACGGCGAAGGCCGCTGGAACTCCCTCGCTGGCTGCGCAGGTCTGAAAAGAACCGGAAAGAGCTGCAGATTACGGTGGCTCAACTATCTCCGCCCCGATGTCCGTCGTGGCAACATCACCCTTGAAGAACAGCTTCTGATTCTTGAGCTTCATTCTCGCTGGGGTAACAGATGGTCAAAAATTGCACAACACTTGCCAGGAAGGACTG

*MdoMYB222*

TTGTCTCCCAGCTCAAACCAGACAAAAGGAAATGGATATTAATAATGTTCATGATCAGATGAGATCAGCACGTTATGGCTCATTAGCAGCAGCAGCAACTCATGATCAACCAATTGAAGGACTGGACTTAAGAAAAGGTCCATGGACAGCCGAGGAAGACTCCATTCTCAACGATTACGTCAACATCCATGGTGAAGGTCGCTGGAATTCCTTGG
