# Supplementary material for: Genome Wide Analysis of the Apple MYB Transcription Factor Family Allows the Identification of MdoMYB121 Gene Confering Abiotic Stress Tolerance in Plants
Source: PLoS One. 2013 Jul 26;8(7):e69955. doi: 10.1371/journal.pone.0069955 (PMC3735319; doi:10.1371/journal.pone.0069955)
Supplement: Text S2 — References for MYB genes which have been functionally characterized in Arabidopsis . (DOC) [file pone.0069955.s010.doc]

**Text S2. References for *MYB* genes which have been functionally characterized in *Arabidopsis*.**

Baumann K, Rodriguez MP, Bradley D, Venail D, Bailey P, et al. (2007) Control of cell and petal morphogenesis by R2R3 MYB transcription factors. Development 134: 1691-1701.

Zhang Y, Cao G, Qu LJ, Gu H (2009) Characterization of *Arabidopsis* MYB transcription factor gene AtMYB17 and its possible regulation by LEAFY and AGL15. J Genet Genomics 36: 99-107.

Jakoby M, Falkenhan D, Mader MT, Brininstool G, Wischnitzki E, et al. (2008) Transcription al profiling of mature *Arabidopsis* trichomes reveals that NOECK encodes the MIXT A-like transcriptional regulator MYB106. Plant Physiol 148: 1583-1602

Gigolashvili T, Engqvist M, Yatusevich R, Müller C, Flügge U, et al. (2007) HAG2/MYB76 and HAG3/MYB29 exert a specific and coordinated control on the regulation of aliphatic glucosinolate biosynthesis in *Arabidopsis thaliana*. New Phytol 177: 627-642

Gigolashvili T, Yatusevich R, Berger B, Müller C, Flügge U, et al. (2007) The R2R3-MYB transcription factor HAG1/MYB28 is a regulator of methionine-derived glucosinolate biosynthesis in *Arabidopsis thaliana*. Plant J 51: 247-261

Gigolashvili T, Berger B, Mock H, Müller C, Weisshaar B, et al. (2007) The transcription factor HIG1/MYB51 regulates indolic glucosinolate biosynthesis in *Arabidopsis thaliana*. Plant J 50: 886-901

Zhou J, Lee C, Zhong R, Ye Z, et al. (2009) MYB58 and MYB63 are transcriptional activators of the lignin biosynthetic pathway during secondary cell wall formation in *Arabidopsis*. Plant Cell 21: 248-266

Cui MH, Yoo KS, Hyoung S, Nguyen HTK, Kim YY, et al. (2013) An *Arabidopsis* R2R3-MYB transcription factor, AtMYB20, negatively regulates type 2C serine/threonine protein phosphatases to enhance salt tolerance. FEBS letters 2013.04.028

Ito T, Nagata N, Yoshiba Y, Ohme-Takagi M, Ma H, et al. (2007) *Arabidopsis MALE STERILITY1* encodes a PHD-Type transcription factor and regulates pollen and tapetum development. Plant Physiol 19: 3549-3562.

Cominelli E, Sala T, Calvi D, Gusmaroli G, Tonelli C, et al. (2008) Over-expression of the *Arabidopsis* AtMYB41 gene alters cell expansion and leaf surface permeability. The Plant J 53: 53-64.

Lippold F, Sanchez DH, Musialak M, Schlereth A, Scheible WR, et al. (2009) AtMYB41 regulates transcriptional and metabolic responses to osmotic stress in Arabidopsis. Plant Physiol 149: 1751-1772.

Denekamp M, Smeekens SC (2003) Integration of wounding and osmotic stress signals determines the expression of the *AtMYB102* transcription factor gene. Plant Physiol 132: 1415-1423.

Vos MD, Denekamp M, Dicke M, Vuylsteke M, Loon LV, et al. (2006) The *Arabidopsis thaliana* transcription factor AtMYB102 functions in defense against the insect herbivore *Pieris rapae*. Plant Signal Behav 1: 305-311.

Li SF, Milliken ON, Pham H, Seyit R, Napoli R, et al. (2009) The *Arabidopsis* MYB5 transcription factor regulates mucilage synthesis, seed coat development, and trichome morphogenesis. Plant Physiol 21: 72-89.

Gonzalez A, Mendenhall J, Huo Y, Lloyd A (2008) TTG1 complex MYBs, MYB5 and TT2, control outer seed coat differentiation. Dev. Biol 325: 412-421.

Hemm MR, Herrmann KM, Chapple C (2001) AtMYB4: a transcription factor general in the battle against UV. Trends Plant Sci 6: 135-136.

Jin H, Cominelli E, Bailey P, Parr A, Mehrtens F, et al. (2000) Transcriptional repression by AtMYB4 controls production of UV-protecting sunscreens in *Arabidopsis.* EMBO J 19: 6150-6161.

Preston J, Wheeler J, Heazlewood J, Li SF, Parish RW (2004) AtMYB32 is required for normal pollen development in *Arabidopsis thaliana*. Plant J 40: 979-995.

Dubos C, Gourrierec J, Baudy A, Huep G, Lanet E, et al. (2008) MYBL2 is a new regulator of flavonoid biosynthesis in *Arabidopsis thaliana*. Plant J 55: 940-953

Zhu J, Verslues P, Zheng X, Lee B, Zhan X, et al. (2005) HOS10 encodes an R2R3-type MYB transcription factor essential for cold acclimation in plants. Proc Natl Acad Sci USA 102: 9966-9971.

Stracke R, Ishihara H, Huep G, Barsch A, Mehrten F, et al. (2007) Differential regulation of closely related R2R3-MYB transcriptionfactors controls flavonol accumulation in different parts of the *Arabidopsis thaliana* seedling. Plant J 50: 660-677.

Petroni K, Falasca G, Calvenzani V, Allegra D, Stolfi C, et al. (2008) The *AtMYB11* gene from *Arabidopsis* is expressed in meristematic cells and modulates growth in planta and organogenesis in vitro. J Exp Bot 59: 1201-1213.

Lepiniec L, Debeaujon L, Routaboul J, Baudry A, Pourcel L, et al. (2006) Genetics and biochemistry of seed flavonoids. Annu Rev. Plant Biol 57: 405-430.

Lai LB, Nadeau JA, Lucas J, Lee EK, Nakagawa T, et al. (2005) The Arabidopsis R2R3 MYB proteins FOUR LIPS and MYB88 restrict divisions late in the stomatal cell lineage. Plant Cell 17: 2754-2767.

Xie Z, Li D, Wang L, Sack FD, Grotewold E (2010) Role of the stomatal development regulators FLP/MYB88 in abiotic stress responses. Plant J 64: 731-739.

Jung C, Seo JS, Han SW, Koo YJ, Kim CH, et al. (2007) Overexpression of *AtMYB44* enhances stomatal closure to confer abiotic stress tolerance in transgenic *Arabidopsis*. Plant Physiol 146: 623-635.

Nguyen XC, Hoang MHT, Kin HS, Lee K, Liu X, et al. (2012) Phosphorylation of the transcriptional regulator MYB44 by mitogen activated protein kinase regulates *Arabidopsis* seed germination. Biochem Biophys Res Commun 423: 703-708.

Park MY, Kang J, Kim SY (2011) [Overexpression of *AtMYB52* confers ABA hypersensitivity and drought tolerance](http://link.springer.com/article/10.1007/s10059-011-0300-7), Mol Cells 31: 447-454.

Lee D, Geisler M, Springer PS (2009) LATERAL ORGAN FUSION1 and LATERAL ORG AN FUSION2 function in lateral organ separation and axillary meristem formation in *Arabidopsis*. Development 136: 2423-2432

Punwani JA, Rabiger DS, Lloyd A, Drews GN (2008) The MYB98 subcircuit of the synergid gene regulatory network includes genes directly and indirectly regulated by MYB98. Plant J 55: 406-414.

Zhang Y, Cao G, Qu L, Gu H (2008) Involvement of an R2R3-MYB transcription factor gene *AtMYB118* in embryogenesis in *Arabidopsis.*

Wang X, Niu Q, Teng C, Li C, Mu J, et al. (2008) Overexpression of *PGA37/MYB118* and *MYB115* promotes vegetative-to-embryonic transition in *Arabidopsis*. Cell Res 19: 224-235.

Zhang Y, Cao G, Qu L, Gu H (2009) Involvement of an R2R3-MYB transcription factor gene AtMYB118 in embryogenesis in *Arabidopsis.* Plant Cell Rep 28: 337-346.

Millar AA, Gubler F (2005) The Arabidopsis *GAMYB-Like* genes, *MYB33* and *MYB65*, are microRNA-regulated genes that redundantly facilitate anther development. Plant Physiol 17: 705-721.

Reyes J, Chua N (2007) ABA induction of miR159 controls transcript levels of two MYB factors during Arabidopsis seed germination. Plant J 49: 592-606.

Mu RL, Cao YR, Liu YF, Lei G, Zou HF, et al. (2009) An R2R3-type transcription factor gene *AtMYB59* regulates root growth and cell cycle progression in *Arabidopsis.* Cell Res 19: 1291-1304.

Devaiah B, Madhuvanthi R, Karthikeyan AS, Raghothama KG (2009) Phosphate starvation responses and gibberellic acid biosynthesis are regulated by the *MYB62* transcription factor in *Arabidopsis.* Mol Plant 2: 43-58.

Mengiste T, Chen X, Salmeron J, Dietrich R (2003) The *BOTRYTIS SUSCEPTIBLE1* gene encodes an R2R3MYB transcription factor protein that is required for biotic and abiotic stress responses in Arabidopsis. Plant Cell 15: 2551-2565.

Shin B, Choi G, Yi H, Yang S, Cho I, et al. (2002) *AtMYB21*, a gene encoding a flower-specific transcription factor, is regulated by COP1. Plant J 30: 23-32.

Yang XY, Li JG, Pei M, Gu H, Chen ZL, et al. (2007) [Over-expression of a flower-specific transcription factor gene AtMYB24 causes aberrant anther development](http://link.springer.com/article/10.1007/s00299-006-0229-z). Plant Cell Rep 26: 219-228.

Zhong R, Richardson EA, Ye ZH (2007)The MYB46 transcription factor is a direct target of SND1 and regulates secondary wall biosynthesis in *Arabidopsis.* Plant Cell 19: 2776-2792.

McCarthy RL, Zhong R, Ye ZH (2009) [MYB83 is a direct target of SND1 and acts redundantly with MYB46 in the regulation of secondary cell wall biosynthesis in Arabidopsis](http://pcp.oxfordjournals.org/content/50/11/1950.short). Plant & Cell Physiol 50: 1950-1964.

Payne CT, Zhang F, Lloyd AM (2000) *GL3* encodes a bHLH protein that regulates trichome development in Arabidopsis through interaction with GL1 and TTG1. Genetics 156: 1349-1362.

Kirik V, Schnittger A, Radchuk V, Adler K, Hülskamp M, et al. (2001) Ectopic expression of the *Arabidopsis* *AtMYB23* gene induces differentiation of trichome cells. Dev Biol 235: 366-377.

Liang YK, Dubos C, Dodd lC, Holroyd GH, Hetherington AM, et al. (2005) AtMYB61, an R2R3-MYB transcription factor controlling stomatal aperture in *Arabidopsis thaliana.* Curr Biol 15: 1201-1206.

Penfield S, Meissner RC, Shoue DA, Carpita NC, Bevan MW (2001) *MYB61* is required for mucilage deposition and extrusion in the *Arabidopsis* seed coat. Plant Cell 13: 2777-2791.

Yang C, Xu Z, Song J, Conner K, Barrena GV, et al. (2007) *Arabidopsis* *MYB26/MALE STERILE35* regulates secondary thickening in the endothecium and is essential for anther dehiscence. Plant Cell 19: 534-548.

Öhman D, Demedts B, Kumar M, Gerber L, Gorzsás A, et al. (2013) MYB103 is required for *FERULATE-5-HYDROXYLASE* expression and syringyl lignin biosynthesis in Arabidopsis stems. Plant J 73: 63-76.

Feng C, Andreasson E, Maslak A, Mock HP, Mattsson O, et al. (2004) *Arabidopsis MYB68* in development and responses to environmental cues. Plant Sci 167: 1099-1107.

Yang F, Wang Q, Schmitz G, Müller D, Theres K (2012) The bHLH protein ROX acts in concert with RAX1 and LAS to modulate axillary meristem formation in Arabidopsis. Plant J 71: 61-70.

Phan HA, Li SF, Parish RW (2011) MYB80, a regulator of tapetal and pollen development, is functinally conserved in crops. Plant Mol Biol 78: 171-183.

Chen Y, Zhang X, Wu W, Chen Z, Gu H, et al. (2006) Overexpression of the wounding-responsive gene *AtMYB15* activates the shikimate pathway in *Arabidopsis.* J Integr Plant Biol 48: 1084-1095.

Zhou J, Lee C, Zhong R, Ye ZH (2009) MYB58 and MYB63 are transcriptional activators of the lignin biosynthetic pathway during secondary cell wall formation in Arabidopsis. Plant Cell 21: 248-266.

Zhong R, Lee C, Zhou J, McCarthy R, Ye ZH (2008) A battery of transcription factors involved in theregulation of secondary cell wall biosynthesis in *Arabidopsis*. Plant Cell 20, 2763-2782.

Vailleau F, Daniel X, Tronchet M, [Montillet](http://www.pnas.org/search?author1=Jean-Luc+Montillet&sortspec=date&submit=Submit)[§](#aff-1) JL, [Triantaphylidès](http://www.pnas.org/search?author1=Christian+Triantaphylid%A8%A8s&sortspec=date&submit=Submit)[§](#aff-1) C, et al. (2002) A R2R3-MYB gene, *AtMYB30*, acts as a positive regulator of the hypersensitive cell death program in plants in response to pathogen attack. Proc Natl Acad Sci USA 99: 10179-10184.

Seo PJ, Xiang F, Qiao M, Park JY, Lee YN, et al. (2009) The MYB96 transcription factor mediates abscisic acid signaling during drought stress response in Arabidopsis. Plant Physiol 151: 275-289.

Seo PJ, Park CM (2010) MYB96-mediated abscisic acid signals induce pathogen resistance response by promoting salicylic acid biosynthesis in Arabidopsis. New Phytol 186: 471- 483.

Seo PJ, Lee SB, Suh MC, Park MJ, Go YS, et al. (2011) The MYB96 transcription factor regulates cuticular wax biosynthesis under drought conditions in *Arabidopsis.* Plant Cell 23: 1138-1152.

Oh JE, Kwon Y, Kim JH, Noh H, Hong SW, et al. (2011) A dual role for MYB60 in stomatal regulation and root growth of *Arabidopsis thaliana* under drought stress. Plant Mol Biol 77: 91-103.

Lin-Wang K, Micheletti D, Palmer J, Volz R, Lozano L, et al. (2011) High temperature reduces apple fruit colour via modulation of the anthocyanin regulatory complex. Plant, Cell & Environ 34: 1176-1190.

Gao JJ, Shen XF, Zhang Z, Peng RH, Xiong AS, et al. (2011) The myb transcription factor *MdMYB46* suppresses anthocyanin in transgenic *Arabidopsis*. Plant Cell Tiss Organ Cult 106: 235-242.

Abe H, Urao T, Ito T, Seki M, Shinozaki K, et al. (2003) *Arabidopsis* AtMYC2 (bHLH) and AtMYB2 (MYB) function as transcriptional activators in abscisic acid signaling. Plant Cell 15: 63-78.

Kirik V, Schnittger A, Radchuk V, Adler K, Hülskamp M, et al. (2001) Ectopic expression of the *Arabidopsis* *AtMYB23* gene induces differentiation of trichome cells. Develop Biol 235: 366-377.

Chen Y, Chen Z, Kang J, Kang D, Gu H (2013) *AtMYB14* regulates cold tolerance in *Arabidopsis*. 31: 87-97.
